# Oral microbiome signatures of post-stroke cognitive impairment

**DOI:** 10.3389/fmicb.2026.1722999

**Published:** 2026-06-05

**Authors:** Jianchao Xu, Yingying Chang, Jiang Ma, Hui Wang, Xiaoyan Li, Xiuming Chen, Shu Niu, Yukun An, Yubin Zhao

**Affiliations:** 1 Department of Rehabilitation Medicine, Shijiazhuang People's Hospital, Shijiazhuang, China; 2 Graduate School, Hebei University of Chinese Medicine, Shijiazhuang, China; 3 Department of Cardiology, The Traditional Chinese Medicine Hospital of Shijiazhuang, Shijiazhuang, China

**Keywords:** 16S/genetics, cognition disorders/diagnosis, exploratory microbial signature, machine learning, mouth/microbiology, ribosomal, RNA, stroke/complications

## Abstract

**Introduction:**

To investigate the characteristics of oral microbiota in patients with post-stroke cognitive impairment (PSCI) and to evaluate its potential as a noninvasive candidate microbial signature associated with PSCI.

**Methods:**

This cross-sectional study enrolled 108 participants, including 40 PSCI patients, 40 post-stroke patients with normal cognition (PSNC), and 28 healthy controls (HC). Saliva samples were collected for 16S rRNA sequencing. Diversity analyses, differential taxa identification, and correlation analyses were performed. Candidate microbial signatures were screened using the least absolute shrinkage and selection operator (LASSO) regression and Random Forest (RF), followed by the construction of multiple machine learning models.

**Results:**

Compared with PSNC and HC, patients with PSCI exhibited significantly reduced richness and diversity of the oral microbiota, and beta-diversity analyses suggested group-level differences in community composition. The relative abundances of gram-negative taxa, such as *Proteobacteria*, *Campylobacterota, Gammaproteobacteria, Pseudomonadales*, and *Alloprevotella* were increased in PSCI samples. In contrast, commensal taxa *Leptotrichia*, and *Veillonella* were markedly decreased. Significant associations were observed between differential taxa and cognitive scores (MMSE and MoCA). Three key microbial features were ultimately identified. Models based on these features showed favorable exploratory internal discriminatory performance between PSCI and PSNC within this dataset. In the test set, the RF model achieved an AUC of 0.979 and an Average Precision of 0.983. However, these findings should be interpreted cautiously and require validation in larger independent cohorts.

**Conclusion:**

Patients with PSCI present with notable oral microbiota dysbiosis, characterized by depletion of commensal taxa and enrichment of Gram-negative bacteria. Oral microbiota-based models showed favorable exploratory internal discriminatory performance within this dataset, but these findings remain hypothesis-generating and require external validation in larger independent cohorts.

## 1 Introduction

Stroke is the second leading cause of death worldwide and represents a major public health burden (GBD 2021 Causes of Death Collaborators, 2024). Survivors frequently experience long-lasting functional deficits. Post-stroke cognitive impairment (PSCI) is a clinical syndrome characterized by cognitive deficits that emerge after the index stroke and persist for more than 3–6 months (Mijajlović et al., 2017). PSCI is highly prevalent. Epidemiological studies report that 24–53.4% of stroke patients develop the condition (Douiri et al., 2013; Lo et al., 2019). Its incidence frequently increases over time, affecting approximately 40% of patients 1 year after stroke (Sexton et al., 2019), and up to 80% by 4 years (Mahon et al., 2017). Compared with patients without cognitive impairment, those with PSCI have a 1.59-fold higher risk of recurrent stroke and a 2.07-fold higher risk of death (Gaynor et al., 2018). Therefore, timely identification and accurate diagnosis of PSCI are critical for improving quality of life and long-term outcomes after stroke.

The pathogenesis of PSCI remains incompletely understood. Its assessment still relies primarily on neuropsychological scales, including the MMSE, MoCA, NINDS-CSN 5-Minute Test, Informant Questionnaire on Cognitive Decline in the Elderly, Rotterdam Cambridge Cognitive Examination, and Oxford Cognitive Screen (Mijajlović et al., 2017; Huang et al., 2022). Multiple instruments are available. However, test performance is sensitive to assessor expertise, patient engagement, and the testing environment, which can introduce subjectivity and bias. Consequently, there is an urgent need for objective approaches to PSCI.

The human body harbors abundant microorganisms across multiple sites, including the gut, skin, lungs, and oral cavities. Adults are estimated to carry approximately 38 trillion bacteria (Sender et al., 2016b), roughly comparable to the number of human cells (Sender et al., 2016a). The oral microbiota is the second largest microbial community in the body (Deo and Deshmukh, 2019). Distinct oral habitats—including saliva, tongue dorsum, tooth surfaces, and gingiva—harbor microbial communities that are broadly similar in taxonomic composition. Across these sites, approximately 50 species are commonly detected, with *Firmicutes*, *Proteobacteria*, *Bacteroidota*, *Actinobacteriota*, and *Fusobacteriota* (Segata et al., 2012). Oral dysbiosis contributes to local diseases, including dental caries, periodontitis, and mucosal disorders (Curtis et al., 2020; Curtis et al., 2011). Because the oral cavity is the initial gateway to the digestive and respiratory tracts, dysbiosis may also promote systemic disease via transient bacteremia (Han and Wang, 2013). Dysbiosis of the oral microbiome has been linked to inflammatory bowel disease, diabetes, obesity, and rheumatoid arthritis (Gao et al., 2018). Oral microbiota changes are associated with several neurological disorders. In Parkinson’s disease, reduced diversity has been observed (Sureda et al., 2020). In Alzheimer’s disease (AD), alterations in the oral microbiota have also been reported (Rozas et al., 2021). In multiple sclerosis, higher abundances of *Staphylococcus*, *Fusobacterium*, *Bacteroides*, *Porphyromonas*, and *Prevotella* have been linked to exacerbated inflammation (Zangeneh et al., 2021). The oral microbiome is closely related to stroke. Changes in oral microbial diversity and structure have been implicated in stroke occurrence (Leonov et al., 2024; Zhong et al., 2024). The presence of oral bacteria in the gut during the acute phase may serve as an early indicator of poor prognosis in acute ischemic stroke (Liang et al., 2025). Additionally, dynamic changes in oral microbial diversity and structure have been shown to predict disease progression and outcomes in stroke-associated pneumonia (Ren et al., 2023). However, the relationship between PSCI and the oral microbiome has not been investigated. To address this gap, the present study characterizes the oral microbiome in Chinese patients with PSCI and evaluates the potential of oral microbial features as candidate microbial signature.

## 2 Materials and methods

### 2.1 Study and participants information

This single-center cross-sectional study enrolled patients with stroke who were admitted for inpatient rehabilitation at Shijiazhuang People’s Hospital (Shijiazhuang, China) from September 2023 to September 2024. Inclusion criteria were: (1) diagnosis of stroke; (2) age 30 and 80 years; (3) time since stroke onset > 3 months; (4) clear consciousness, hemodynamic stability, good compliance, and ability to complete cognitive assessments. Exclusion criteria were: (1) cognitive impairment attributable to other causes (e.g., Alzheimer’s disease, Parkinson’s disease, brain tumor, metabolic or infectious disorders); (2) severe comorbidities such as malignant neoplasms; (3) inability to complete neuropsychological testing because of aphasia, acute delirium or other disturbances of consciousness, or psychiatric disorders; (4) pregnancy or lactation; (5) history of severe allergy or presence of autoimmune disease; (6) acute infectious disease within the previous 2 weeks; (7) use of antibiotics, proton pump inhibitors, systemic corticosteroids, or probiotic therapy within the previous 3 months; and (8) failure to obtain a saliva sample during hospitalization.

Eligible stroke participants underwent cognitive evaluation by trained cognitive-rehabilitation therapists. Based on performance on the MMSE and MoCA, patients were classified into a post-stroke normal cognition (PSNC) group or a PSCI group. Cognitive status was classified using predefined MMSE/MoCA-based criteria. For the MoCA, 1 point was added for participants with ≤12 years of formal education, and a corrected score <26 was considered abnormal, in accordance with the original MoCA scoring recommendations and official scoring instructions (Nasreddine et al., 2005). For the MMSE, education-adjusted Chinese cutoffs were applied (≤17 for illiterate participants, ≤20 for primary school education, and ≤24 for junior high school education or above), consistent with prior studies in Chinese post-stroke populations (Qu et al., 2015). Education was used only for cognitive-score correction according to the predefined MMSE and MoCA scoring rules. In this study, patients were classified as PSCI if either the education-corrected MoCA score or the education-adjusted MMSE score was below the corresponding cutoff. Patients whose MMSE and MoCA scores were both within the normal range were classified as PSNC. In parallel, age-, sex-, and body mass index–matched healthy volunteers without stroke were recruited from the hospital’s health examination center and assigned to the healthy control (HC) group.

### 2.2 Clinical data and sample collection

All post-stroke participants were consecutively recruited during inpatient rehabilitation and were in the chronic phase (>3 months after stroke onset) at the time of clinical assessment and saliva sampling. Upon study enrollment, rehabilitation physicians with standardized training collected demographic and clinical information. Validated clinical assessments—including the Mini-Mental State Examination (MMSE), Montreal Cognitive Assessment (MoCA), National Institutes of Health Stroke Scale (NIHSS), Fugl-Meyer Assessment (FMA), Berg Balance Scale (BBS), and Barthel Index (BI)—were administered on the morning of the second day after enrollment.

Saliva samples were collected on the same morning (the second day after enrollment) to ensure temporal consistency between clinical assessment and microbiome profiling. To further characterize disease stage, the interval from stroke onset to sampling was recorded for each participant. Participants were instructed to maintain routine oral hygiene prior to sampling. Before saliva collection, each participant fasted for at least 2 h and rinsed the mouth with purified water 30 min prior to sampling. Approximately 5 mL of saliva was expectorated into a sterile collection tube. Within 1 h of collection, samples were snap-frozen in liquid nitrogen for 5–10 min and then transferred to a −80 °C freezer for long-term storage. Although saliva collection procedures were standardized, detailed oral health information, including periodontal status, dental caries, tooth loss, salivary flow, dental prostheses, recent dental treatment, and detailed oral hygiene practices, was not systematically collected.

### 2.3 16S rRNA sequencing

To reduce potential batch-related technical, all saliva samples were submitted together and processed as a single sequencing batch using the same DNA extraction protocol, PCR amplification procedure, library preparation kit, sequencing platform, and bioinformatic workflow. All samples were included in the same sequencing project and sequenced in the same Illumina NovaSeq run. Total genome DNA from samples was extracted using Cetrimonium Bromide (CTAB) method (Allen et al., 2006). DNA concentration and purity was monitored on 1% agarose gels. According to the concentration, DNA was diluted to 1 ng/μL using sterile water. The V3–V4 hypervariable region of the bacterial 16S rRNA gene was amplified using primers 341F (5′-CCTAYGGGRBGCASCAG-3′) and 806R (5′-GGACTACNNGGGTATCTAAT-3′). Reactions contained 15 μL Phusion^®^ High-Fidelity PCR Master Mix (New England Biolabs), 2 μM of each primer, and ~10 ng template DNA. Thermal cycling consisted of an initial denaturation at 98 °C for 1 min; 30 cycles of 98 °C for 10 s, 50 °C for 30 s, and 72 °C for 30 s; followed by a final extension at 72 °C for 5 min. PCR products were mixed 1:1 with 1 × loading buffer containing SYBR Green and separated on a 2% agarose gel for detection products was mixed in equidensity ratios. Then, mixture PCR products were purified with Qiagen Gel Extraction Kit (Qiagen, Germany). Sequencing libraries were generated using TruSeq® DNA PCR-Free Sample Preparation Kit (Illumina, United States) following manufacturer’s recommendations and index codes were added. The library quality was assessed on the Qubit@ 2.0 Fluorometer (Thermo Scientific) and Agilent Bioanalyzer 2100 system. At last, the library was sequenced on an Illumina NovaSeq platform and 250 bp paired-end reads were generated. However, no separate extraction blank, PCR negative control, or mock community was included in the present sequencing batch.

### 2.4 Sequence data processing

Paired-end reads were assigned to samples based on their unique barcode and truncated by cutting off the barcode and primer sequence. Quality filtering on the raw tags was performed under specific filtering conditions to obtain the high-quality clean tags according to the fastp. Paired-end reads were merged using FLASH (Magoč and Salzberg, 2011), a very fast and accurate analysis tool, which was designed to merge paired-end reads when at least some of the reads overlap the read generated from the opposite end of the same DNA fragment. The tags were compared with the Silva database using UCHIME Algorithm (Edgar et al., 2011) to detect chimera sequences, and then the chimera sequences were removed (Haas et al., 2011). Then the Effective Tags finally obtained. Amplicon sequence variant (ASV) was analyzed by Deblur, which uses error profiles to obtain putative error-free sequences from Illumina sequencing platform. For each representative sequence, the Silva Database (Quast et al., 2013) was used based on Mothur algorithm to annotate taxonomic information. Multiple sequence alignment of all ASV representative sequences was performed using MAFFT software (Katoh et al., 2002) to construct a phylogenetic tree. To eliminate sequencing depth bias, data normalization was carried out across all samples based on the sample with the lowest sequencing depth. Subsequent alpha and beta diversity analyses were conducted using the normalized dataset.

### 2.5 Bioinformation analysis

Sequence data were processed and analyzed using QIIME and R software (version 4.4.2). Alpha diversity was assessed to evaluate the within-sample microbial richness and evenness using four indices: Chao1, Shannon, Simpson, and ACE. Rarefaction curves, rank abundance curves, and species accumulation curves were generated using the *vegan* package in R to visualize species richness and sampling depth. Inter-group comparisons of alpha diversity indices were performed using Student’s *t*-test or Wilcoxon rank-sum test for two groups, and Tukey’s test or Kruskal–Wallis’s test for multiple groups, depending on data distribution. Ternary plots were constructed using the *ternaryplot* function from the *vcd* package to depict the compositional structure of dominant taxa across samples. Venn diagram analysis was performed to visualize the shared and unique ASVs among different groups. The analysis was conducted using the *VennDiagram* package.

Beta diversity was calculated to assess between-sample differences in microbial community composition. Principal coordinates analysis (PCoA) based on the Bray–Curtis distance was performed using the *phyloseq* package to visualize community dissimilarity (Ren et al., 2013). Group-level differences in community composition were formally tested using PERMANOVA (adonis2) and ANOSIM, implemented in the *vegan* package. To determine whether the observed differences were driven by heterogeneous dispersion rather than centroid separation, the homogeneity of multivariate dispersion was further assessed using the betadisper function in the *vegan* package.

Taxonomic classification was conducted at multiple hierarchical levels, including phylum, class, order, family, and genus. Differences in microbial composition between two groups were identified using the Wilcoxon rank-sum test, while comparisons among more than two groups were performed using the Kruskal–Wallis test followed by Dunn’s post-hoc test for pairwise comparisons. To identify key taxa with significant intergroup differences, linear discriminant analysis effect size (LEfSe) (Khleborodova et al., 2024) was applied and taxa with log10 Linear Discriminant Analysis (LDA) scores > 4 were considered significantly discriminative. Spearman correlation analysis was performed to evaluate the associations between the oral microbiota and clinical indicators. For analyses involving multiple comparisons (taxonomic comparisons, LEfSe analysis, and correlation analysis), *p* values were adjusted using the Benjamini–Hochberg (BH) false discovery rate (FDR) procedure, and FDR-adjusted *p* values (*q* values) < 0.05 were considered statistically significant.

For machine-learning analyses, only PSCI and PSNC samples were included. To avoid information leakage, the machine-learning pipeline was performed independently of the full-dataset differential abundance analyses. The differential abundance analyses described above were used for biological interpretation only and were not used to restrict the initial candidate feature pool for model development. Starting from the genus-level relative-abundance table, the PSCI and PSNC samples were first randomly divided into a training set and a held-out test set at a ratio of 7:3 using stratified sampling according to outcome status. The test set was not used for feature filtering, feature selection, preprocessing parameter estimation, hyperparameter tuning, threshold selection, or model fitting. Feature filtering was performed exclusively within the training set. Only genus-level taxa with resolved annotations were retained, while unidentified, unclassified, uncultured, unknown, ASV/OTU placeholder annotations, and higher-level taxonomic placeholders were removed. Taxa were further retained if they had a prevalence ≥10%, a mean relative abundance ≥0.1% in the training set. LASSO logistic regression (Fu et al., 2025) was conducted using the *glmnet* package to identify candidate microbial features within the training set. In parallel, a Random Forest (RF) model was built using the *randomForest* package, with hyperparameters tuned by out-of-bag (OOB) error and variable importance summarized as the mean decrease in Gini impurity. Taxa with a Gini coefficient greater than 1 were then selected as candidate taxa. The intersection of features selected by LASSO and RF was then used to define the final candidate microbial signature for internal discrimination analyses.

Based on these features, eight supervised machine learning algorithms were applied to construct classification models. Logistic regression (LR) (LaValley, 2008) using the glm function from the *stats* package. K-nearest neighbors (KNN) (Zhang, 2016) using the kknn method from the *caret* package. Support vector machine (SVM) (Valkenborg et al., 2023) using the svm function from the *e1071* package. Extreme gradient boosting (XGBoost) (Ma et al., 2020) using the *xgboost* package. Random forest (RF) (Hu and Szymczak, 2023) models were fitted with the *randomForest* package, with the optimal *mtry* parameter selected by cross-validation. Decision tree models (TREE) (Mehrpour et al., 2023) were implemented with the *rpart* package, with complexity parameter tuned by cross-validation. Light gradient boosting machine (LightGBM) (Zhang et al., 2019) was trained using the *lightgbm* package. Neural networks (NNET) (Zhang et al., 2023) were constructed with the *neuralnet* package. Hyperparameter tuning was performed exclusively within the training set using internal cross-validation procedures (primarily 10-fold cross-validation, with model-specific settings for boosting algorithms), while the held-out test set was reserved solely for final evaluation. Nested cross-validation was not implemented, and therefore the reported performance estimates should be interpreted as internal rather than fully unbiased estimates of generalizability.

Model performance was evaluated using the *pROC* package to generate receiver operating characteristic (ROC) curves and calculate the area under the curve (AUC) values. Precision–Recall (P–R) curves were plotted for each model with the precision axis limited to 0.5–1, and the Average Precision (AP) was calculated and reported for each model using the *PRROC* package. The 95% confidence intervals for AUC and AP were estimated by using 2,000 bootstrap resampling iterations. In addition, clinically relevant classification metrics were calculated in the test set, including sensitivity, specificity, accuracy, balanced accuracy, positive predictive value, negative predictive value, and F1 score. Probabilistic prediction performance and calibration-related performance were assessed using the Brier score and logarithmic loss. Model interpretability was further explored using Shapley Additive Explanations (SHAP), which provided global feature importance rankings and beeswarm visualizations. To evaluate the internal stability of model performance, repeated cross-validation was performed within the training set for the model showing the best overall performance in the test set, considering AUC, AP, classification metrics, and calibration-related metrics. In addition, bootstrap resampling was conducted within the training set to assess the stability of selected microbial taxa. Nested cross-validation was not implemented, and no external validation cohort was available. Therefore, all machine-learning results were interpreted strictly as exploratory internal discrimination rather than evidence of clinical diagnostic utility or externally validated candidate microbial signature performance.

### 2.6 Statistical analysis

All statistical analyses and data visualizations were performed using R software (version 4.4.2), SPSS (version 27.0, IBM Corp., Armonk, NY, United States), and GraphPad Prism (version 10.0). Continuous variables were expressed as mean ± standard deviation (SD) or median with interquartile range (IQR), depending on data distribution. Group comparisons were conducted using Student’s *t*-test, non-parametric tests (e.g., Wilcoxon rank-sum test), or one-way analysis of variance (ANOVA), as appropriate. Categorical variables were presented as counts and percentages, and comparisons were made using the Chi-square test. Education level was compared among groups at baseline and was incorporated into the predefined MMSE/MoCA scoring correction. Therefore, education was not automatically included as an additional covariate in the primary microbiome analyses unless a relevant baseline imbalance was observed. Covariate-adjusted analyses were performed within the stroke cohort, with covariates selected based on their clinical variables and their distribution between the PSCI and PSNC groups in baseline comparisons. A two-sided *p* value < 0.05 was considered statistically significant.

## 3 Results

### 3.1 Demographic and clinical data

**Figure 1:**
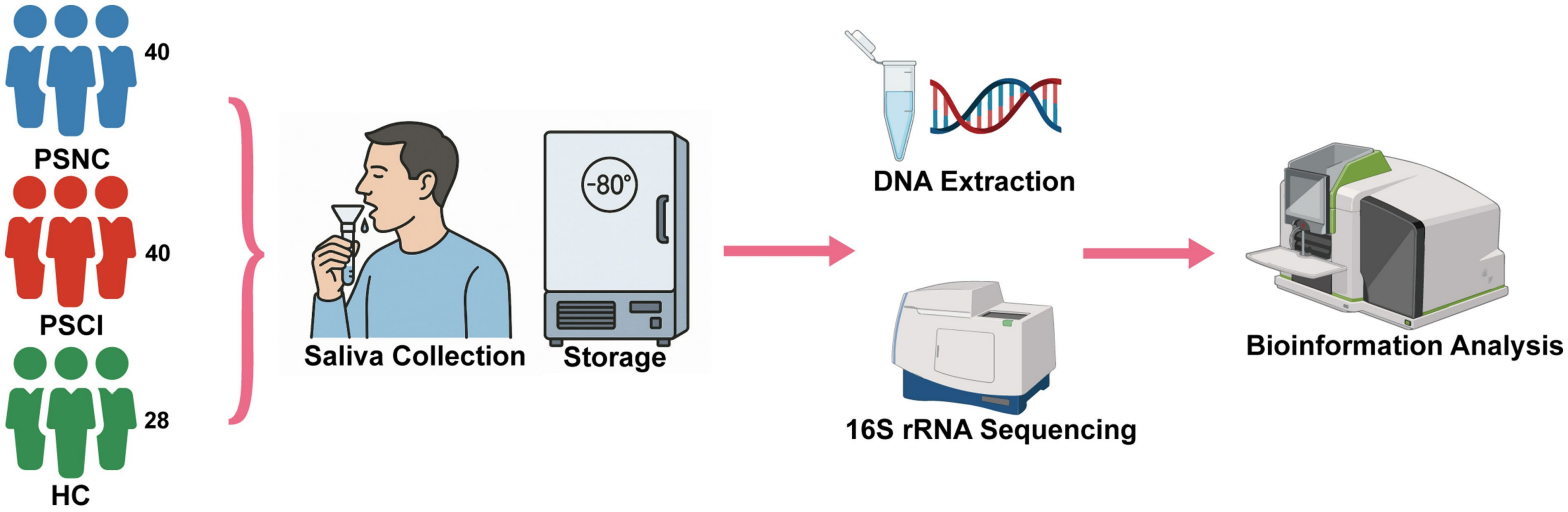
Flow chart of participant enrollment and study design.

Based on the predefined inclusion and exclusion criteria, a total of 108 participants were enrolled (Figure 1). Demographic and clinical characteristics of each group are summarized in Table 1. Saliva samples were obtained from 40 patients with PSCI (mean age, 57.30 ± 14.73 years; men, 31 [77.50%]) and 40 patients with PSNC (mean age, 58.20 ± 12.73 years; men, 29 [72.50%]). The time from stroke onset to enrollment (stroke duration) was 189.25 ± 25.62 days in the PSCI group and 179.30 ± 23.11 days in the PSNC group, with no statistically significant difference between groups (*p* = 0.052), suggesting no major between-group difference in disease stage at the time of sampling. Education level was comparable among the PSCI and PSNC (*p* = 0.934) and three groups (*p* = 0.996).

**Table 1:** Characteristics of the study participants.

Characteristics		PSCI (*n* = 40)	PSNC (*n* = 40)	HC (*n* = 28)	*P* value
Gender	Female	9 (22.50%)	11 (27.50%)	10 (35.70%)	0.606^*^/0.488^#^
	Male	31 (77.50%)	29 (72.50%)	18 (64.30%)	
Stroke type	Infraction	31 (77.50%)	26 (65.00%)	/	0.217^*^
	Hemorrhage	9 (22.50%)	14 (35.00%)	/	
Education	Primary	15 (37.50%)	15 (37.50%)	10 (35.70%)	0.934^*^/0.996^#^
	Secondary	21 (52.50%)	20 (50.00%)	15 (53.60%)	
	University	4 (10.00%)	5 (12.50%)	3 (10.70%)	
Age, years		57.30 ± 14.73	58.20 ± 12.73	59.00 ± 5.68	0.771^*^/0.831^#^
Height, m		1.69 ± 0.09	1.68 ± 0.08	1.67 ± 0.08	0.783^*^/0.643^#^
Weight, kg		72.55 ± 11.88	70.95 ± 12.04	72.39 ± 11.18	0.551^*^/0.826^#^
BMI, kg/m^2^		25.31 ± 2.79	24.87 ± 2.89	25.78 ± 1.46	0.497^*^/0.628^#^
SBP, mmHg		139.73 ± 18.68	141.87 ± 24.07	132.32 ± 11.05	0.657^*^/0.098^#^
DBP, mmHg		90.80 ± 13.48	89.30 ± 14.79	87.93 ± 9.94	0.637^*^/0.553^#^
Heart Rate		85.10 ± 15.31	83.80 ± 16.58	82.18 ± 11.89	0.717^*^/0.659^#^
Stroke duration, days		189.25 ± 25.62	179.30 ± 23.11	/	0.052^*^
Drunk, *n* (%)		17 (42.50%)	22 (55.00%)	15 (53.60%)	0.263^*^/0.972^#^
Smoke, *n* (%)		28 (70.00%)	20 (50.00%)	18 (64.30%)	0.068^*^/0.130^#^
Hypertension, *n* (%)		30 (75.00%)	32 (80.00%)	8 (28.57%)	0.592^*^/ < 0.001^#^
Diabetes, *n* (%)		15 (37.50%)	10 (25.00%)	6 (21.43%)	0.228^*^/ < 0.001^#^
Cardiovascular disease, *n* (%)		11 (27.50%)	13 (32.50%)	3 (10.71%)	0.626^*^/ < 0.001^#^
Hyperlipemia, *n* (%)		14 (35.00%)	12 (30.00%)	3 (10.71%)	0.633^*^/ < 0.001^#^
MMSE		18.15 ± 2.63	28.70 ± 1.29	/	< 0.001^*^
MoCA		12.60 ± 4.49	28.20 ± 1.69	/	< 0.001^*^
NIHSS		13.80 ± 3.57	9.40 ± 1.81	/	< 0.001^*^
Fugl-Meyer		20.42 ± 6.59	21.10 ± 4.08	/	0.828^*^
Berg		6.80 ± 3.56	11.40 ± 4.31	/	< 0.001^*^
Barthel Index		29.10 ± 13.31	36.63 ± 16.50	/	0.032^*^

BMI, body mass index; SBP, Systolic Blood Pressure; DBP, Diastolic Blood Pressure; MMSE, Mini-Mental State Examination; MoCA, Montreal Cognitive Assessment; NIHSS, National Institutes of Health Stroke Scale. ^#^PSCI vs PSNC vs HC; ^*^PSCI vs PSNC.

In cognitive assessments, the PSCI group performed significantly worse than the PSNC group on both the MMSE and MoCA (both *p* < 0.001). BBS scores significantly differed between groups (*p* < 0.05). NIHSS scores were higher in PSCI, indicating more severe neurological impairment (*p* < 0.001). BI scores were lower in PSCI (*p* = 0.032), reflecting poorer activities of daily living. In addition, 28 age- and sex-matched healthy controls (HC) [mean age, 59.00 ± 5.68 years; men, 18 (64.30%)] voluntarily provided saliva samples for sequencing analysis.

### 3.2 Sequencing results

A total of 11,193,693 raw reads were obtained from 108 saliva samples, with an average of 103,645 raw reads per sample. After quality control, 11,065,017 clean reads were retained, including 4,113,925 reads from the PSCI group, 4,119,407 from the PSNC group, and 2,831,685 from the HC group. Following chimera filtering, 9,749,701 effective reads remained, comprising 3,524,560 from PSCI, 3,727,464 from PSNC, and 2,497,677 from HC. On average, effective reads accounted for 87.22% of total reads per sample.

**Figure 2:**
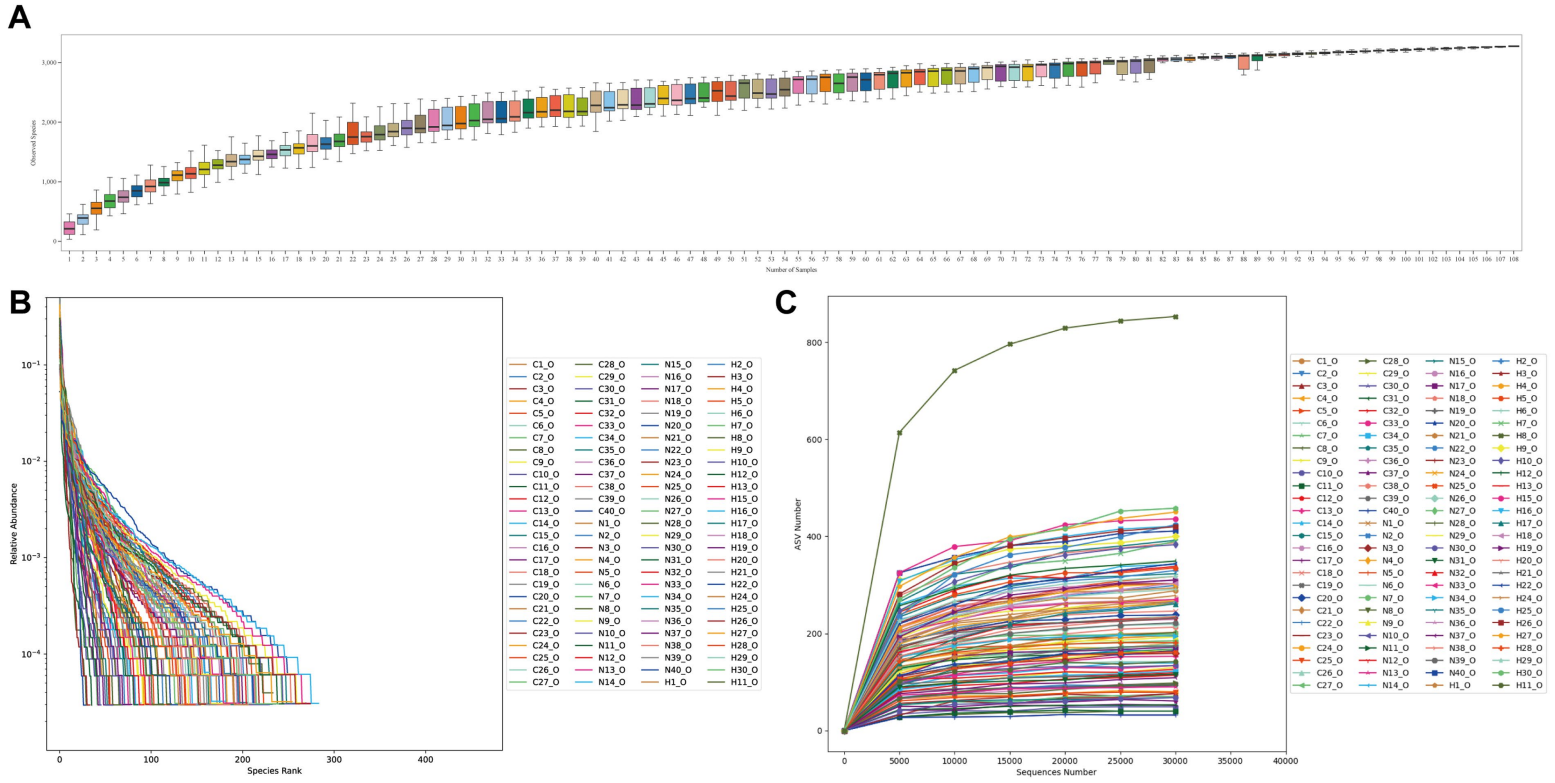
Curves of microbial diversity. **(A)** Species accumulation boxplot. **(B)** Rank abundance curve. **(C)** Rarefaction curve.

Rarefaction curves extended rightward along the x-axis and approached a plateau, indicating that sequencing depth was sufficient for downstream analyses (Figure 2C). Rank abundance curves also exhibited rightward extension with a flattening trend, suggesting high species richness and evenness across samples (Figure 2B). Species accumulation boxplots demonstrated that the number of newly observed species plateaued after approximately 89 samples, reflecting adequate sampling depth (Figure 2A).

### 3.3 The decreased oral microbial diversity in PSCI

**Figure 3:**
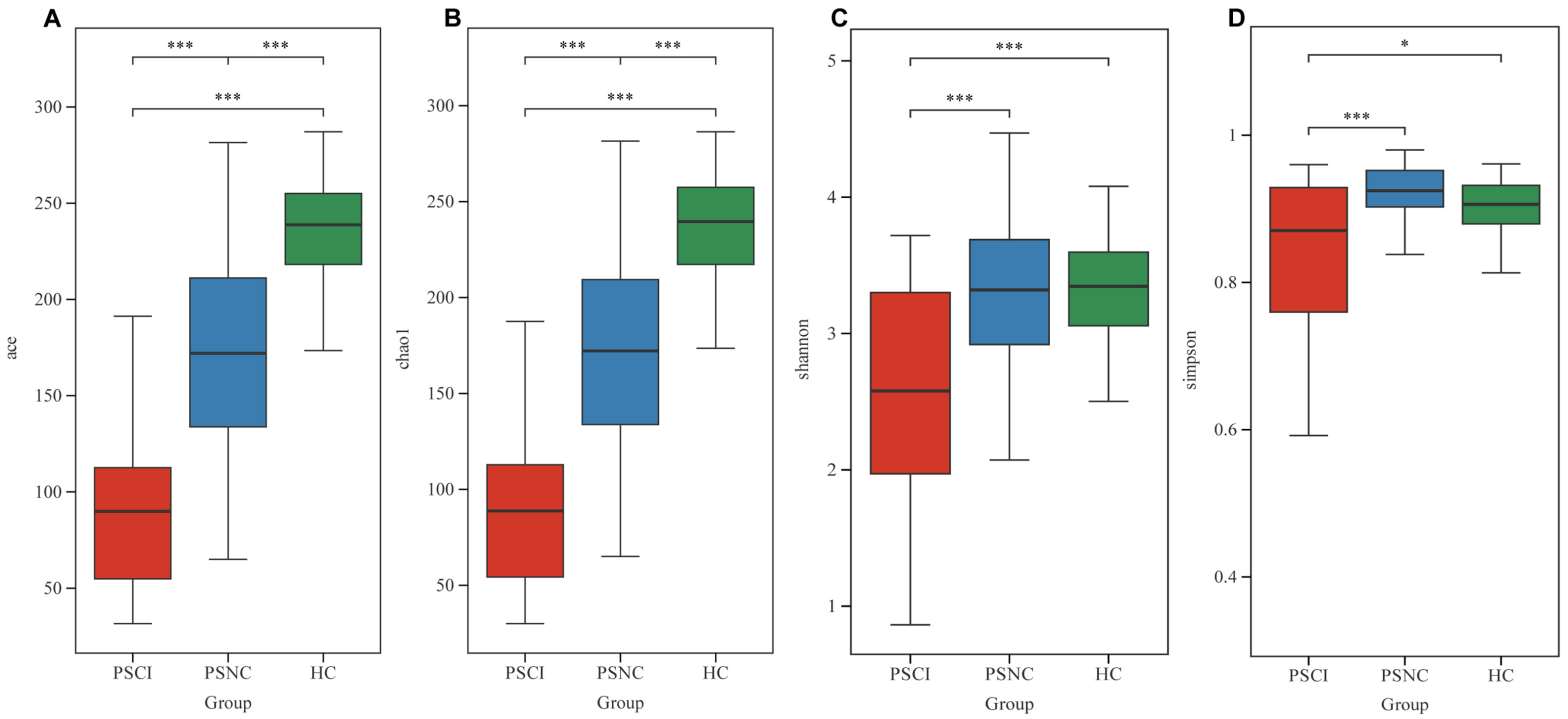
Alpha diversity indices among the three groups: **(A)** ACE index; **(B)** Chao1 index; **(C)** Shannon index; **(D)** Simpson index. Boxplots show the median and interquartile range (IQR). Group differences were assessed using the Kruskal–Wallis test, followed by Dunn’s *post hoc* pairwise comparisons. ^*^p < 0.05, ^***^p < 0.001.

Alpha diversity indices, including ACE, Chao1, Shannon, and Simpson, revealed significant differences among the three groups (Figure 3). The PSCI group exhibited markedly reduced richness (ACE: 93.15 ± 46.38; Chao1: 92.52 ± 46.02) and diversity (Shannon: 2.56 ± 0.78; Simpson: 0.81 ± 0.16) compared to the PSNC and HC groups (all *p* < 0.05). Based on baseline comparisons, NIHSS, BBS, and BI were selected as covariates for analyses within the stroke cohort. After covariate adjustment, significant differences in alpha diversity between PSCI and PSNC remained, with PSCI showing lower Shannon diversity (β = −0.666, 95% CI −1.198 to −0.134, *p* = 0.0148), lower Simpson index (β = −0.098, 95% CI −0.194 to −0.002, *p* = 0.0457), and reduced richness for both Chao1 (β = −69.757, 95% CI −109.149 to −30.365, *p* < 0.001) and ACE (β = −69.855, 95% CI −109.283 to −30.427, *p* < 0.001) (Supplementary Table S1). In contrast, no significant differences were observed between the PSNC and HC groups in the Shannon and Simpson indices. These results indicate reduced oral microbial richness and diversity in patients with PSCI.

### 3.4 Differences between PSCI and control individuals in the oral microbiota

**Figure 4:**
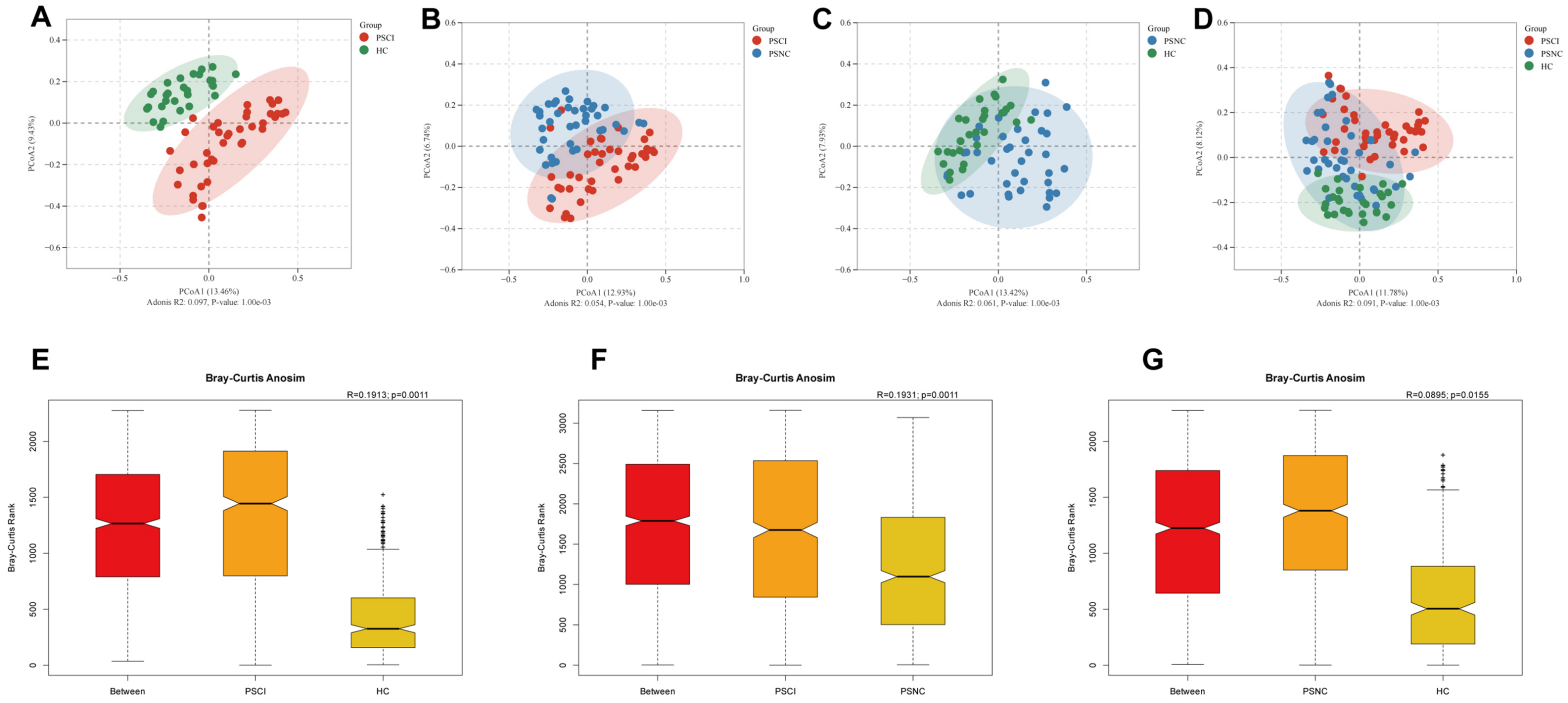
Beta diversity comparisons based on Bray–Curtis distances. **(A–D)** PCoA plots illustrating differences in community structure: **(A)** PSCI vs. HC, **(B)** PSCI vs. PSNC, **(C)** PSNC vs. HC, and **(D)** all three groups. Each point represents one sample, and the percentage of variance explained by each axis is shown. The PERMANOVA (adonis) results (R^2^ and *p* values) are reported in each panel. **(E–G)** ANOSIM results based on Bray–Curtis dissimilarity for the corresponding pairwise comparisons: **(E)** PSCI vs. HC, **(F)** PSCI vs. PSNC, and **(G)** PSNC vs. HC. Higher *R* values indicate greater between-group separation.

To compare microbial community composition across groups, beta diversity was evaluated based on the Bray–Curtis’s distance. PERMANOVA revealed significant differences in microbial structure between the PSCI and PSNC groups (*R*^2^ = 0.054, *p* < 0.001; Figure 4B), PSCI and HC groups (*R*^2^ = 0.097, *p* < 0.001; Figure 4A), and PSNC and HC groups (*R*^2^ = 0.061, *p* < 0.001; Figure 4C). A significant difference was also observed when comparing all three groups (*R*^2^ = 0.091, *p* < 0.001; Figure 4D). However, these *R*^2^ values suggest modest effect sizes, indicating that group status explains a limited proportion of the overall variance in oral community composition. After covariate adjustment within the stroke cohort, PERMANOVA based on Bray–Curtis distance remained significant between PSCI and PSNC (*R*^2^ = 0.0199, *F* = 1.632, *p* = 0.015; Supplementary Table S2).

Additionally, ANOSIM analysis was performed to further determine whether between-group variation exceeded within-group variation. Significant differences were observed between PSCI and HC (*R* = 0.1913, *p* = 0.0011; Figure 4E), PSNC and HC (*R* = 0.0895, *p* = 0.0155; Figure 4G), and PSCI and PSNC (*R* = 0.1931, *p* = 0.0011; Figure 4F). These results suggest that between-group differences exceeded within-group variability, although the effect sizes were modest. To assess whether PERMANOVA results could be driven by heterogeneous within-group variability, the homogeneity of multivariate dispersion was tested using PERMDISP (betadisper). No significant difference in dispersion was detected for Bray–Curtis distances (*F* = 1.893, *p* = 0.167), supporting that the PERMANOVA findings reflect differences in community composition rather than dispersion artifacts (Supplementary Table S3).

### 3.5 Composition of the oral microbiota in PSCI and control groups

**Figure 5:**
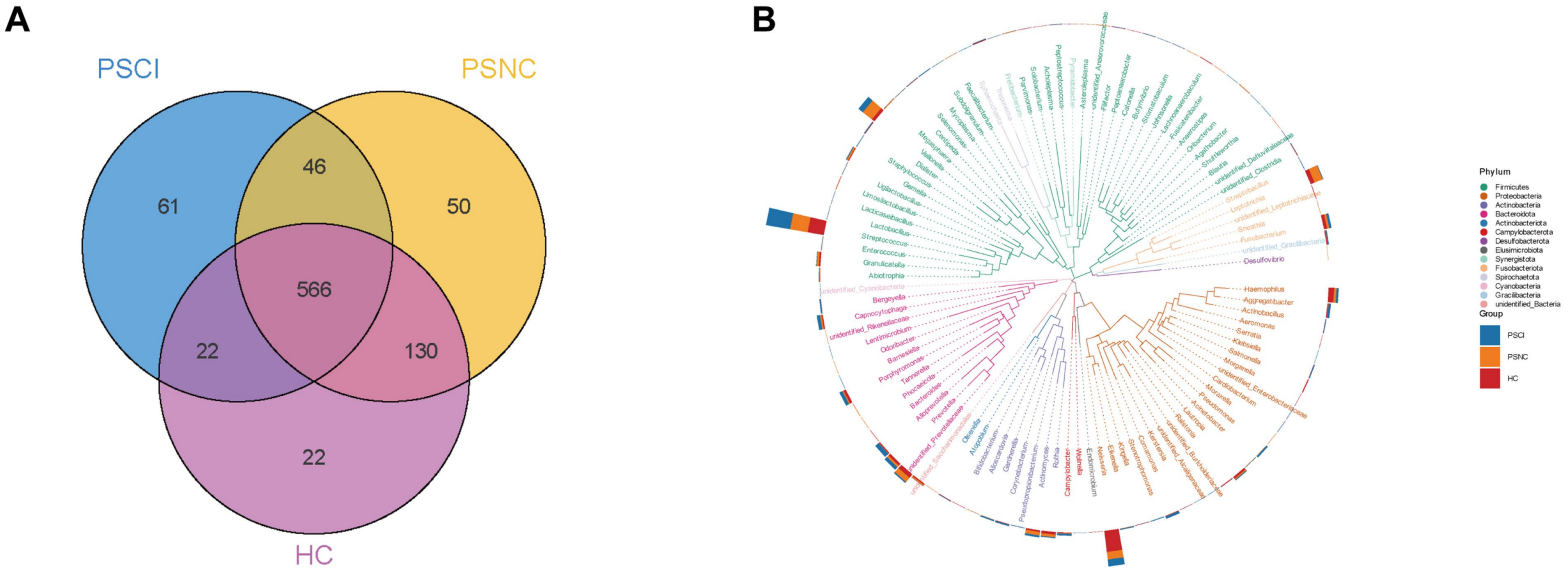
ASV overlaps and taxonomic phylogeny at the genus level. **(A)** Venn diagram showing shared and unique ASVs among the three groups. **(B)** Phylogenetic tree based on representative ASV sequences at the genus level. ASV, Amplicon sequence variant.

A total of 3,274 amplicon sequence variants (ASVs) were identified across the 108 saliva samples. Venn diagram analysis revealed that 566 ASVs were shared among all three groups. Pairwise comparisons showed that 612 ASVs were shared between the PSCI and PSNC groups, 588 between PSCI and HC, and 696 between PSNC and HC. Unique ASVs were also detected, with 61 specific to PSCI, 50 to PSNC, and 22 to HC (Figure 5A). Taxonomic annotation of the ASVs at the genus level indicated that the dominant taxa across all groups primarily included *Streptococcus*, *Neisseria*, *Veillonella*, *Leptotrichia*, *Haemophilus*, *Prevotella*, *Fusobacterium*, and *Actinomyces* (Figure 5B).

**Figure 6:**
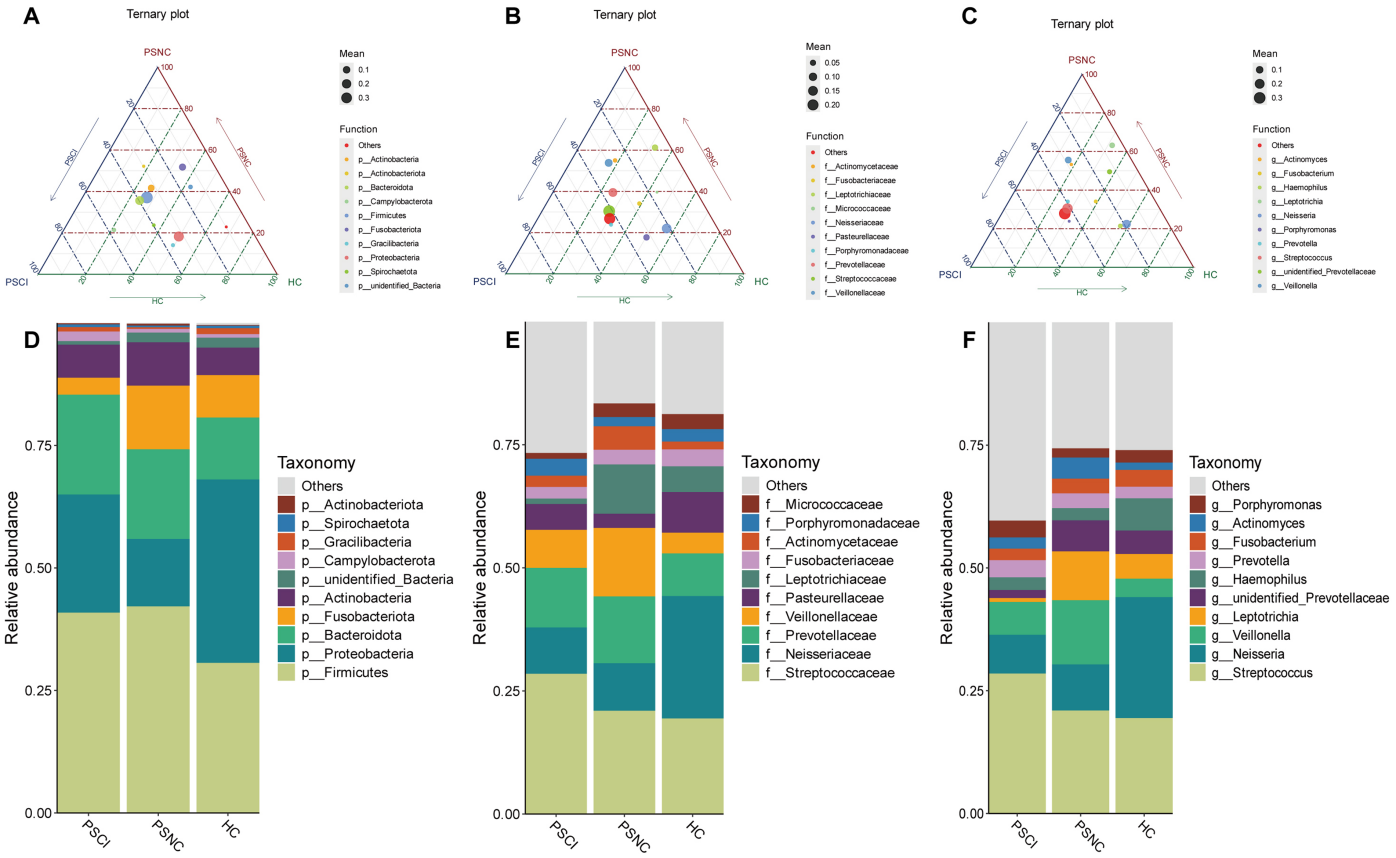
Composition and distribution of dominant microbial taxa across groups. **(A–C)** Ternary plots of the top 10 taxa at the phylum **(A)**, family **(B)**, and genus **(C)** levels. Each point represents a taxon, positioned according to relative abundance across PSCI, PSNC, and HC groups. Circle size indicates mean relative abundance. **(D–F)** Stacked bar plots of the top 10 taxa at the phylum **(D)**, family **(E)**, and genus **(F)** levels. “Others” includes taxa outside the top 10.

At the phylum level (Figures 6A,D), the dominant taxa in the PSCI group were *Firmicutes* (40.88%), *Proteobacteria* (24.09%), *Bacteroidota* (20.40%), *Actinobacteria* (6.75%), and *Fusobacteriota* (3.45%), together comprising 95.56% of the total community. In the PSNC group, the leading phyla included *Firmicutes* (42.15%), *Bacteroidota* (18.29%), *Proteobacteria* (13.76%), *Fusobacteriota* (13.00%), and *Actinobacteria* (8.83%), accounting for 96.04% of the overall composition. The HC group was predominantly composed of *Proteobacteria* (37.42%), *Firmicutes* (30.62%), *Bacteroidota* (12.66%), *Fusobacteriota* (8.67%), and *Actinobacteria* (5.60%), with these taxa representing 94.96% of the microbial community.

At the family level (Figures 6B,E), the top taxa in the PSCI group were *Streptococcaceae* (28.48%), *Prevotellaceae* (12.12%), *Neisseriaceae* (9.40%), *Veillonellaceae* (7.71%), *Pasteurellaceae* (5.26%), *Porphyromonadaceae* (3.45%), *Fusobacteriaceae* (2.35%), *Actinomycetaceae* (2.30%), *Micrococcaceae* (1.17%), and *Leptotrichiaceae* (1.10%). In the PSNC group, *Streptococcaceae* (20.96%) remained the most abundant, followed by *Veillonellaceae* (13.91%), *Prevotellaceae* (13.56%), *Leptotrichiaceae* (10.01%), *Neisseriaceae* (9.66%), *Actinomycetaceae* (4.76%), *Fusobacteriaceae* (2.99%), *Pasteurellaceae* (2.90%), *Micrococcaceae* (2.78%), and *Porphyromonadaceae* (1.87%). In the HC group, the most prevalent families included *Neisseriaceae* (24.84%), *Streptococcaceae* (19.42%), *Prevotellaceae* (8.68%), *Pasteurellaceae* (8.24%), *Leptotrichiaceae* (5.24%), *Veillonellaceae* (4.21%), *Fusobacteriaceae* (3.42%), *Micrococcaceae* (3.06%), *Porphyromonadaceae* (2.53%), and *Actinomycetaceae* (1.59%).

At the genus level (Figures 6C,F), the PSCI group was mainly dominated by *Streptococcus* (28.48%), followed by *Neisseria* (7.90%), *Veillonella* (6.69%), *Prevotella* (3.49%), *Porphyromonas* (3.45%), *Haemophilus* (2.58%), *Fusobacterium* (2.35%), *Actinomyces* (2.28%), and *Leptotrichia* (0.78%). The PSNC group exhibited high abundances of *Streptococcus* (20.96%), *Veillonella* (13.07%), *Leptotrichia* (9.96%), *Neisseria* (9.38%), *Actinomyces* (4.30%), *Prevotella* (3.02%), *Fusobacterium* (2.99%), *Haemophilus* (2.49%), and *Porphyromonas* (1.87%). In the HC group, *Neisseria* (26.02%) and *Streptococcus* (19.42%) were the dominant genera, followed by *Haemophilus* (6.56%), *Leptotrichia* (5.02%), *Veillonella* (3.76%), *Fusobacterium* (3.42%), *Porphyromonas* (2.53%), *Prevotella* (2.35%), and *Actinomyces* (1.50%).

### 3.6 Comparison of the oral microbiota in PSCI and control groups

**Figure 7:**
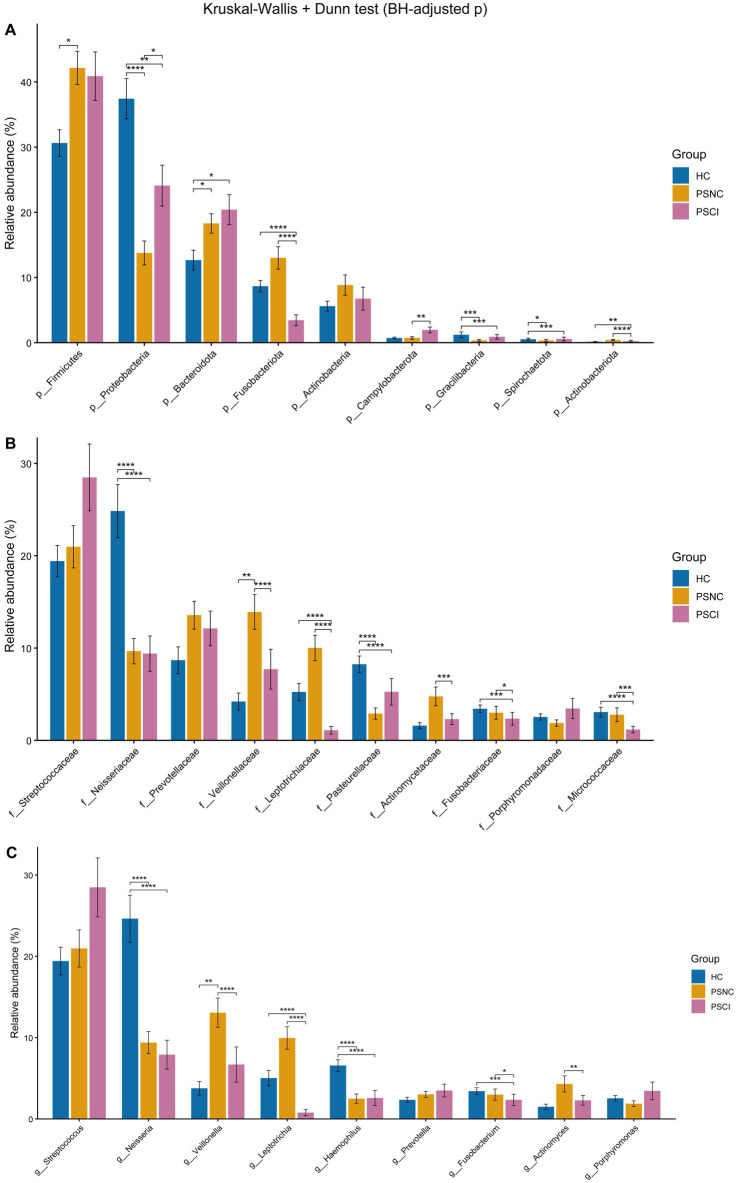
Differential abundance of dominant taxa among the three groups. **(A)** Phylum level. **(B)** Family level. **(C)** Genus level. Bars represent relative abundance (%). Group differences were assessed using the Kruskal–Wallis test followed by Dunn’s *post-hoc* pairwise comparisons, with Benjamini–Hochberg correction for multiple testing. Asterisks denote significance based on BH-adjusted *p* values (*q* values): *q < 0.05, **q < 0.01, ***q < 0.001.

Using the Kruskal–Wallis test followed by Dunn’s post-hoc pairwise comparisons with BH correction, we compared the relative abundance of dominant taxa across groups (Figure 7). The PSCI group showed a clear compositional shift relative to PSNC and HC. In the PSCI vs. PSNC comparison, BH-adjusted analyses identified consistent taxonomic differences across multiple levels. At the phylum level (Figure 7A), *Fusobacteriota* and *Actinobacteriota* were significantly depleted in PSCI, whereas *Proteobacteria* and *Campylobacterota* were enriched (all *q* < 0.05). At the family level (Figure 7B), *Veillonellaceae, Leptotrichiaceae, Actinomycetaceae, Fusobacteriaceae,* and *Micrococcaceae* were significantly reduced in PSCI (all *q* < 0.05), while *Streptococcaceae* showed an increasing trend without reaching significance. At the genus level (Figure 7C), *Veillonella, Leptotrichia, Fusobacterium,* and *Actinomyces* were significantly decreased in PSCI (all *q* < 0.05), whereas *Streptococcus* and *Porphyromonas* were higher in PSCI but not statistically significant.

**Figure 8:**
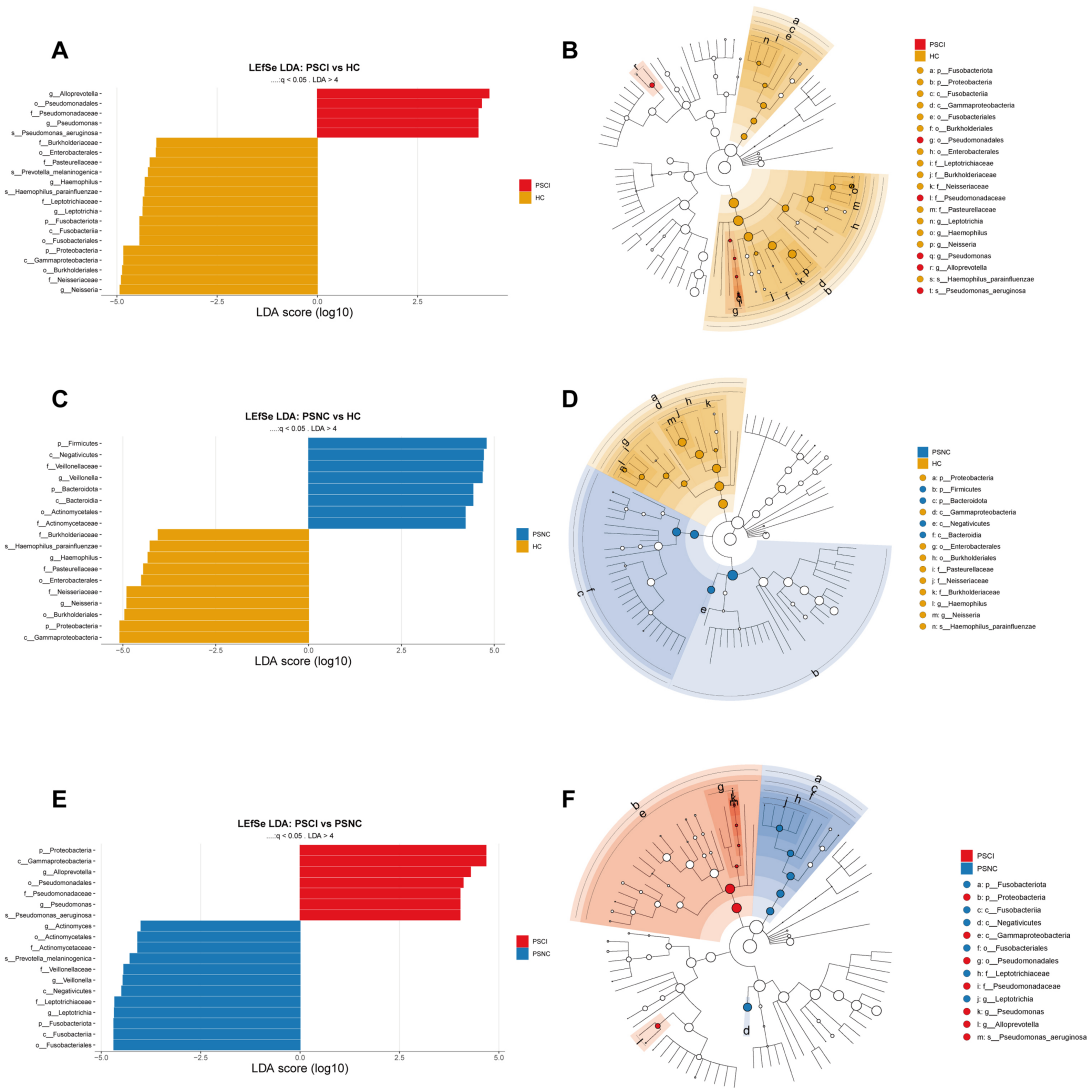
LEfSe analysis of the oral microbiota among groups. **(A,B)** PSCI vs. HC, **(C,D)** PSNC vs. HC, and **(E,F)** PSCI vs. PSNC. **(A,C,E)** LDA score bar plots showing differentially enriched taxa identified by LEfSe, using a significance threshold of BH-adjusted *p* value (*q* value) < 0.05 and log10 LDA score > 4. **(B,D,F)** Cladograms depicting the taxonomic distribution of the discriminative features. Concentric circles represent taxonomic levels from phylum (innermost) to species (outermost). Each node denotes a taxon, with node size proportional to relative abundance; taxa enriched in each group are highlighted in group-specific colors, whereas non-significant taxa are shown in a neutral color. LEfSe, linear discriminant analysis effect size; LDA, linear discriminant analysis.

LefSe analysis was performed to identify the key microbial taxa differentially enriched among the three groups. To reduce the risk of false positives due to multiple comparisons, significance was determined based on BH–adjusted *p* values (*q* < 0.05). In PSCI vs. HC (Figures 8A,B), PSCI was enriched in the *Pseudomonadales–Pseudomonadaceae–Pseudomonas* lineage, including *Pseudomonas aeruginosa*, as well as *Alloprevotella*. In contrast, HC was enriched in taxa spanning the *Fusobacteriota/Proteobacteria* lineages, including *Fusobacteriales* and *Leptotrichiaceae/Leptotrichia*, together with *Neisseriaceae/Neisseria* and *Pasteurellaceae/Haemophilus* (including *Haemophilus parainfluenzae*) and *Prevotella melaninogenica*. In PSNC vs. HC (Figures 8C,D), PSNC showed enrichment of *Firmicutes/Bacteroidota*-associated taxa, notably *Negativicutes* and *Veillonellaceae/Veillonella*, along with *Actinomycetales/Actinomycetaceae*, whereas HC was enriched in *Proteobacteria*-related taxa, including *Gammaproteobacteria*, *Enterobacterales/Burkholderiales*, and *Neisseriaceae/Neisseria* and *Pasteurellaceae/Haemophilus*. In PSCI vs. PSNC (Figures 8E,F), PSCI was enriched in *Proteobacteria*, driven by *Gammaproteobacteria* and the *Pseudomonadales–Pseudomonadaceae–Pseudomonas* lineage (including *P. aeruginosa*) and *Alloprevotella*. Conversely, PSNC was enriched in taxa predominantly from the *Fusobacteriota* and *Firmicutes* lineages, including *Fusobacteriales/Fusobacteriaceae, Leptotrichiaceae/Leptotrichia, Veillonellaceae/Veillonella, Actinomycetales/Actinomycetaceae/Actinomyces,* and *Prevotella melaninogenica* (all *q* < 0.05).

### 3.7 Relationship between oral microbiota and clinical data

**Figure 9:**
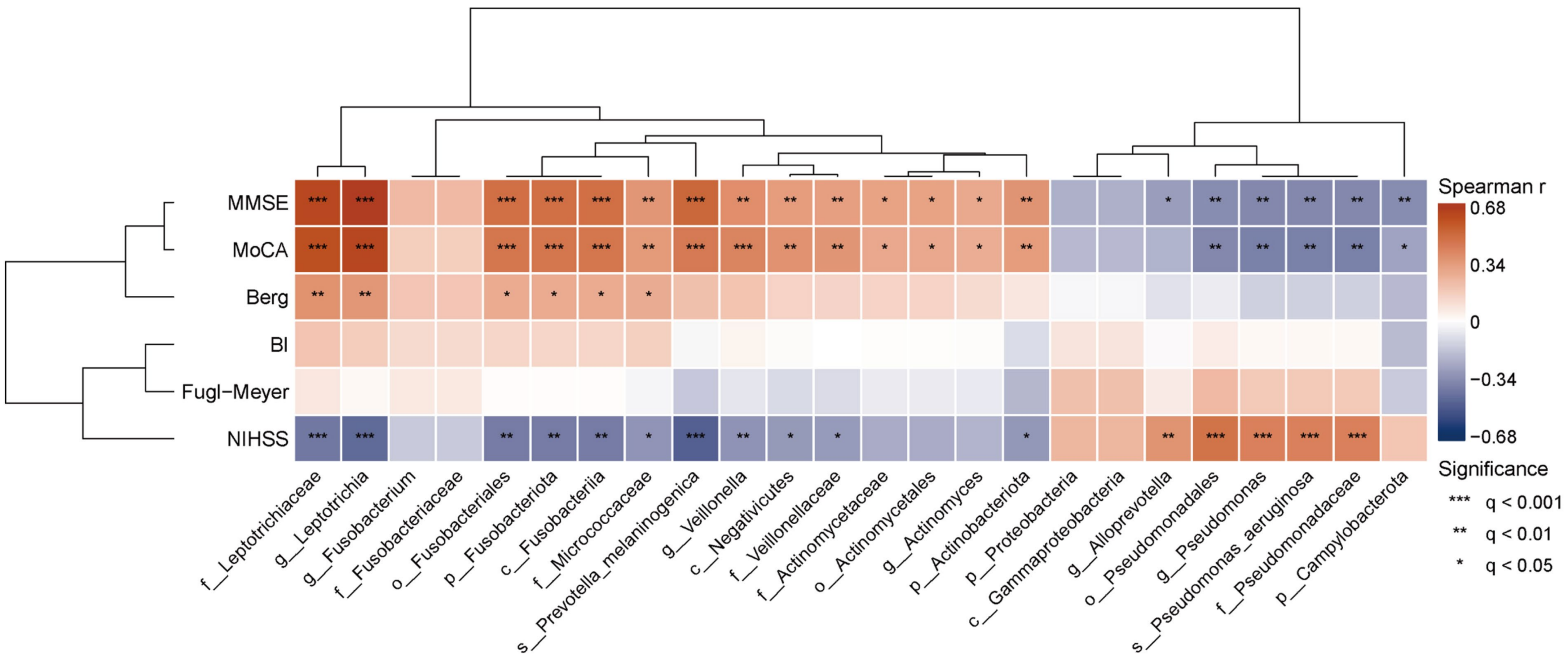
Correlation between oral microbiota and clinical data. Heatmap showing Spearman’s rank correlations (*r*) between differential taxa and clinical scale scores. Colors indicate the direction and magnitude of correlations (red, positive; blue, negative). Statistical significance was evaluated using two-sided Spearman correlation tests with Benjamini–Hochberg correction for multiple comparisons; asterisks denote significance based on FDR-adjusted *p* values (*q* values): *q < 0.05, **q < 0.01, ***q < 0.001. MMSE, Mini-Mental State Examination; MoCA, Montreal Cognitive Assessment; NIHSS, National Institutes of Health Stroke Scale; BBS, Berg Balance Scale; BI, Barthel Index; FMA, Fugl–Meyer Assessment.

Based on relative abundance comparisons and LEfSe analysis, 24 oral taxa were identified as significantly different between the PSCI and PSNC groups. Spearman correlation analysis was subsequently performed to evaluate the associations between these taxa and clinical scores. Several taxa showed significant correlations with cognitive function after BH correction (Figure 9). Among the 24 taxa, 20 were significantly associated with MMSE or MoCA scores.

*Fusobacteriota*-related taxa, including *Leptotrichiaceae/Leptotrichia*, *Fusobacteriaceae/Fusobacterium*, *Fusobacteriales*, and *Fusobacteriia*, as well as *Veillonellaceae/Veillonella*, *Micrococcaceae*, and *Prevotella melaninogenica*, were positively correlated with cognitive performance (all *q* < 0.05). In contrast, *Proteobacteria*-associated taxa, including *Proteobacteria*, *Gammaproteobacteria*, *Pseudomonadales/Pseudomonadaceae*, *Pseudomonas* (particularly *Pseudomonas aeruginosa*), *Alloprevotella*, and *Campylobacterota*, were negatively correlated with MMSE or MoCA scores (all *q* < 0.05).

### 3.8 A three-taxon oral signature shows favorable internal discrimination between PSCI and PSNC

**Figure 10:**
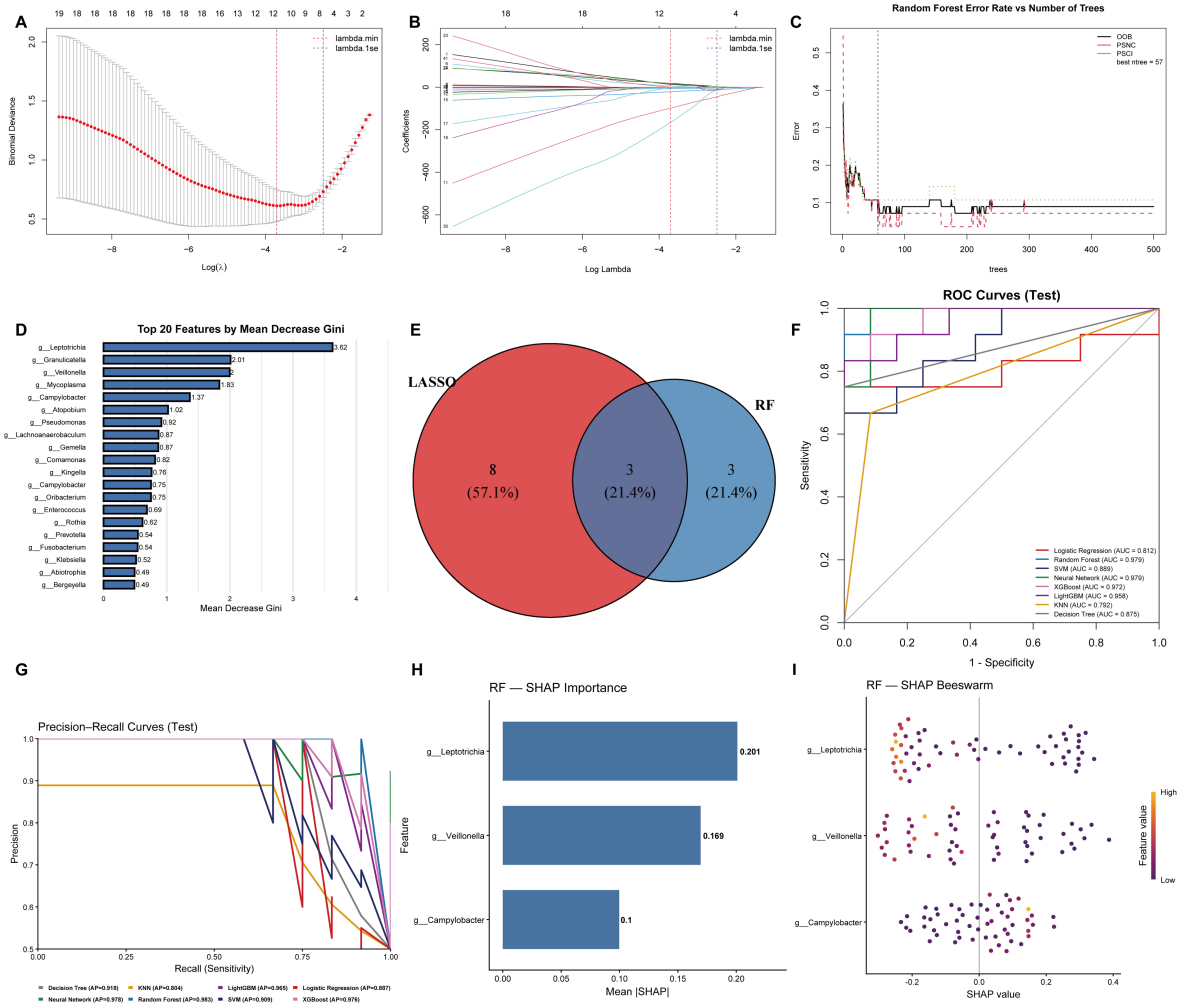
Feature selection, model benchmarking, and interpretability for PSCI internal discrimination using oral microbiota. **(A)** Ten-fold cross-validation for LASSO logistic regression. Red points indicate mean binomial deviance across folds and gray bars indicate ±1 SE. Vertical dashed lines mark *λ*_min (minimum deviance) and λ_1se (1-SE rule). **(B)** LASSO coefficient profiles as a function of log(λ); numbers on the top axis indicate the number of non-zero coefficients at each λ. **(C)** Random forest training curve showing out-of-bag (OOB) error and class-specific errors versus the number of trees (best ntree = 57). **(D)** Top taxa ranked by random forest importance (mean decrease in Gini). **(E)** Overlap of features retained by LASSO and random forest; three shared taxa were selected as a candidate microbial signature for PSCI discrimination. **(F)** ROC curves on the test set for eight classifiers; AUC values are reported in the legend, and the diagonal line indicates random performance. **(G)** Precision–recall curves on the test set; AP denotes average precision. **(H)** Global SHAP importance for the RF model summarized as mean absolute SHAp value (mean|SHAP|). **(I)** SHAP beeswarm plot for the RF model. Each dot represents one sample; the x-axis indicates the SHAP value (feature contribution), and color encodes the corresponding feature value (low to high). LASSO, least absolute shrinkage and selection operator; RF, random forest; OOB, out-of-bag; LR, logistic regression; TREE, decision tree; KNN, *k*-nearest neighbors; SVM, support vector machine; LGBM, light gradient boosting machine; NNET, neural network; XGB, extreme gradient boosting; PR, precision–recall; AP, average precision.

For machine-learning analyses, the 80 stroke patients were stratified into a training set of 56 participants (28 PSCI and 28 PSNC) and a test set of 24 participants (12 PSCI and 12 PSNC). LASSO retained 11 features at Lambda_min (0.024665) and 6 at Lambda_1se (0.082666) (Figures 10A,B). RF achieved the lowest and most stable OOB error at ntree = 57 (Figure 10C), and the Gini importance ranking highlighted a compact set of informative taxa (Figure 10D). Intersecting both methods produced three key microbial features: *Veillonella*, *Leptotrichia* and *Campylobacter* (Figure 10E).

In the test set, the RF model showed favorable exploratory internal discriminatory performance, with an AUC of 0.979 (95% CI: 0.917–1.000) and an AP of 0.983 (95% CI: 0.931–1.000) (Figures 10F,G). The RF model also achieved a sensitivity of 0.917, specificity of 1.000, accuracy of 0.958, balanced accuracy of 0.958, PPV of 1.000, NPV of 0.923, and F1 score of 0.957, with a Brier score of 0.076 and LogLoss of 0.256. The complete performance metrics of all machine-learning models are summarized in Supplementary Table S4. Based on its overall performance, RF was selected for model interpretation. Global SHAP analysis ranked *Leptotrichia* as the dominant contributor, followed by *Veillonella* and *Campylobacter* (Figure 10H). The SHAP beeswarm plot further illustrated the feature effects on individual predictions, indicating that variation in these taxa, particularly *Leptotrichia*, contributed substantially to the predicted probability of PSCI (Figure 10I).

Repeated cross-validation within the training set supported the internal stability of the RF model, with a mean AUC of 0.960 ± 0.052 and a mean AP of 0.969 ± 0.038. Bootstrap stability analysis further showed that the selected taxa were consistently retained, particularly *Leptotrichia* and *Veillonella*, which showed high LASSO, RF, and intersection selection frequencies. Detailed results are provided in Supplementary Tables S5, S6.

## 4 Discussion

Using 16S rRNA gene amplicon sequencing, we characterized the oral microbiota of patients with PSCI, PSNC, and HC. Overall, the oral microbiota of patients with PSCI exhibited oral microbiota alterations, reflected by reduced richness and diversity together with altered community structure relative to PSNC and HC. Several differentially abundant taxa were further associated with cognitive performance, suggesting a potential association between oral microbial patterns and post-stroke cognitive impairment. In addition, exploratory machine-learning analyses suggested that oral microbial features had internal discriminatory potential for distinguishing PSCI from PSNC within this dataset. Collectively, these findings suggest that oral microbiota alterations are associated with PSCI and may have exploratory value for distinguishing cognitive impairment after stroke.

With respect to alpha diversity, ACE, Chao1, Shannon, and Simpson indices were significantly lower in PSCI, indicating reduced richness and diversity of the oral microbiota in this group. Beta-diversity analyses further revealed statistically significant, although modest, differences in overall community structure between PSCI and the comparison groups. Consistent with this, PERMDISP based on Bray–Curtis distances did not detect significant differences in dispersion, suggesting that the PERMANOVA findings were unlikely to be driven by heterogeneous within-group variability. Importantly, after adjustment for selected clinical covariates within the stroke cohort, the differences in both alpha diversity and beta diversity between PSCI and PSNC remained statistically significant, suggesting that these microbial alterations were not fully attributable to differences in neurological severity or functional status alone. Although studies specifically examining oral microbiota alterations in PSCI remain limited, reduced oral microbial diversity and compositional shifts have also been reported in AD (Wu et al., 2021; Liu et al., 2019), which is consistent with our findings. Together, these observations support a potential association between oral microbiota dysregulation and cognitive impairment.

Taxonomic profiling showed that PSCI was characterized by a distinct oral microbial pattern, with enrichment of several taxa commonly linked to inflammatory or disease-associated states, including *Porphyromonas*, *Streptococcus*, *Campylobacterota*, and *Bacteroidota*-related lineages, and depletion of putative commensal taxa such as *Leptotrichia*, *Veillonella*, *Neisseria*, and *Fusobacterium*. In parallel, LEfSe analysis further identified enrichment of *Proteobacteria*, particularly *Gammaproteobacteria* and *Pseudomonas*-related lineages, as well as *Alloprevotella* in PSCI. Taken together, these findings suggest that PSCI is associated with oral dysbiosis characterized by depletion of potentially protective commensals and enrichment of Gram-negative taxa.

*Leptotrichia* are common oral commensals that have been linked to ecological stability and mucosal immune homeostasis (Couturier et al., 2012; Langfeldt et al., 2014). In gestational diabetes, oral dysbiosis is characterized by an enrichment of periodontitis-associated taxa (e.g., *Prevotella*) and a concomitant reduction in *Leptotrichia* (Corrêa et al., 2023). Prior studies suggest that *Leptotrichia* participate in carbohydrate fermentation and may influence local pH balance (Eribe and Olsen, 2008), and may also modulate epithelial inflammatory responses by regulating both pro- and anti-inflammatory cytokines (Eribe and Olsen, 2017). In our study, *Leptotrichia* was depleted in PSCI, which may reflect a shift in oral ecological balance. However, the mechanistic relevance of *Leptotrichia* to PSCI requires validation in longitudinal and functional studies.

*Veillonella* is a common oral commensal frequently observed in healthy oral microbiomes (Tett et al., 2021). It ferments lactic acid to acetate and propionate, lowering local acidity and helping prevent oral disease (Giacomini et al., 2023; Ng and Hamilton, 1971; Delwiche et al., 1985; Bradshaw and Marsh, 1998; Washio et al., 2014). In our study, *Veillonella* abundance was significantly decreased in PSCI, paralleling the overall reduction in community richness. This depletion may indicate a shift toward a less stable oral community. However, whether reduced *Veillonella* contributes to systemic inflammatory signaling or neurocognitive outcomes in PSCI requires further longitudinal and mechanistic validation.

In our study, several gram-negative taxa were enriched in PSCI and negatively correlated with cognitive performance, including *Campylobacterota*, *Proteobacteria* (notably *Gammaproteobacteria*), *Pseudomonas*-related lineages (*Pseudomonadales*, *Pseudomonadaceae*, *Pseudomonas, Pseudomonas aeruginosa*), and *Alloprevotella*. Previous studies have suggested that expansion of *Proteobacteria* may represent a microbial signature of dysbiosis across several inflammatory and metabolic diseases (Rizzatti et al., 2017; Litvak et al., 2017). From a biological perspective, Gram-negative bacteria can provide a source of lipopolysaccharide (LPS), which has been shown in experimental and clinical contexts to activate innate immune pathways such as TLR4–NF-κB and to promote inflammatory responses (Raetz and Whitfield, 2002; Brown and Heneka, 2024). Prior experimental studies have also linked systemic inflammatory stimulation, blood–brain barrier disruption, microglial activation, and neuroinflammation to cognitive dysfunction (Skrzypczak-Wiercioch and Sałat, 2022; Lecca et al., 2022; Brown and Heneka, 2024; Sandiego et al., 2015; Ma et al., 2024). These mechanisms provide a plausible biological framework for future studies. However, our study did not measure circulating LPS, inflammatory cytokines, blood–brain barrier integrity, microglial activation, or other neuroinflammatory markers. Therefore, the enrichment of Gram-negative taxa in PSCI should be interpreted as an observed association, and any involvement of LPS-related systemic inflammation or neuroinflammatory pathways remains hypothetical.

We also observed a directional increase in *Streptococcus* and *Porphyromonas* in the PSCI group, although these genus-level differences did not reach statistical significance in the pairwise abundance comparison. Both taxa are strongly influenced by local oral ecological conditions. *Streptococcus* is an early colonizer in dental biofilm formation (Listgarten, 1994), whereas *Porphyromonas* is closely related to periodontal inflammation and can produce gingipains that degrade periodontal tissues and modulate host immune responses (Reyes, 2021). Therefore, the observed directional increases may reflect oral ecological imbalance, underlying periodontal status, dental caries, oral hygiene differences, or other local oral factors, rather than PSCI-specific microbial changes. Previous studies have linked *Porphyromonas* and related oral pathogens to cognitive impairment and neuroinflammatory processes (Li et al., 2024; Nonaka and Nakanishi, 2020). However, because detailed oral health examinations were not performed in our cohort, we could not determine whether these taxa were associated with PSCI independently of oral disease burden. Accordingly, the findings related to *Streptococcus* and *Porphyromonas* should be interpreted as exploratory and hypothesis-generating.

To explore the internal discriminatory potential of oral microbial features, we constructed machine-learning classifiers to distinguish PSCI from PSNC. Feature selection using LASSO combined with RF yielded an exploratory candidate panel comprising *Veillonella*, *Leptotrichia* and *Campylobacter*. Within this dataset, RF showed the best internal performance, achieving an AUC of 0.979 and an AP value of 0.983. Overall, these findings suggest that oral microbial features may have exploratory value for the internal discrimination of PSCI from PSNC, and that more flexible nonlinear models may better capture complex microbiome–phenotype relationships in this setting.

However, these findings should not be interpreted as evidence of clinical applicability. The study was based on a single-center cohort with an internal 70/30 train–test split and lacked external validation, limiting assessment of generalizability across populations and clinical settings. In addition, although feature selection and hyperparameter tuning were confined to the training set and cross-validation was used to mitigate overfitting, nested cross-validation was not performed. Therefore, the reported performance should be interpreted as internal and may still be optimistic, particularly given the relatively small sample size and high-dimensional microbial data. Further validation in larger, multicenter, and prospectively collected cohorts is required before any clinical translation can be considered.

Beyond local oral effects, an emerging concept is the oral–gut–brain axis (Cho M. Y. et al., 2025), whereby oral microbes and their products may influence gut microbial ecology and downstream neuroimmune signaling. Increasing evidence suggests that oral dysbiosis may be linked to gut microbial perturbations, and that these changes could contribute to systemic inflammation and neuroinflammatory processes (Sansores-España et al., 2021). Oral bacteria are continuously swallowed and may contribute to gut community shifts (Arimatsu et al., 2014), while microbial components such as LPS and inflammatory mediators can promote systemic immune activation (Mohammad and Thiemermann, 2020; Jørgensen et al., 2016). In the context of immune dysregulation and compromised blood–brain barrier integrity, such systemic inflammatory cues may facilitate microglial activation and amplify neuroinflammatory cascades, ultimately contributing to cognitive decline (Adil et al., 2025). Similar concepts have been discussed in neurological disorders such as Alzheimer’s disease, Parkinson’s disease, ischemic stroke, and migraine (Narengaowa et al., 2021; Clasen et al., 2025; Zhong et al., 2024; Cho S. et al., 2025). However, despite this growing body of work in related neurological contexts, a systematic discussion and dedicated investigation of the oral–gut–brain axis specifically in PSCI remains limited. Future PSCI-focused studies incorporating concurrent oral and stool sampling, together with longitudinal cognitive assessments, are needed to clarify cross-site microbial interactions and their potential clinical relevance.

This study has several limitations. First, this was a single-center, cross-sectional study with a modest sample size, which precludes causal inference and limits the representativeness and generalizability of the findings. Therefore, we cannot determine whether oral microbiota dysbiosis is a cause, consequence, or concomitant feature of PSCI, and the dataset may not fully capture the broad spectrum of PSCI-associated oral microbiota alterations. Prospective multicenter longitudinal studies with repeated oral sampling and concurrent cognitive assessments are needed to clarify temporal relationships and validate these findings.

Second, the machine-learning analyses were based on internal validation only. Although feature selection, preprocessing, hyperparameter tuning, and model fitting were performed exclusively within the training set, nested cross-validation was not implemented, and no external validation cohort was available. Thus, the reported model performance may still be optimistic and should be interpreted as exploratory internal discrimination rather than evidence of clinical applicability.

Third, several potential confounders were not comprehensively assessed. Although sample collection procedures were standardized, individual oral hygiene behaviors, circadian rhythm, recent food intake, dietary patterns, and concomitant medications may have influenced oral microbial composition. Detailed oral health information, including periodontal status, dental caries, tooth loss, salivary flow, dental prostheses, recent dental treatment, and structured oral hygiene practices, was not systematically collected. This is particularly relevant because taxa such as *Porphyromonas* and *Streptococcus* are closely related to oral ecological conditions and periodontal status. Therefore, the observed microbial differences may partly reflect unmeasured oral disease burden, oral hygiene differences, medication exposure, or other local and systemic factors rather than PSCI-specific microbial alterations. Future studies should incorporate standardized oral examinations, periodontal indices, caries assessment, tooth-loss records, salivary flow measurements, prosthesis status, recent dental treatment history, structured dietary assessments, and comprehensive medication inventories, followed by multivariable adjustment and pre-specified sensitivity or stratified analyses.

Fourth, although all samples were submitted together and processed as a single sequencing batch using the same experimental and bioinformatic workflow, extraction blanks, PCR negative controls, and mock communities were not included. Therefore, potential low-level reagent contamination, laboratory contamination, sequencing bias, or other unmeasured technical variation cannot be completely excluded. Future studies should prospectively record batch variables, include negative controls and mock communities, and randomize samples across laboratory steps when multiple batches are required.

Finally, 16S rRNA sequencing has limited taxonomic resolution and provides only indirect functional inference compared with shotgun metagenomics. Future studies with larger cohorts should integrate shotgun metagenomics, metatranscriptomics, and metabolomics to better characterize the functional relevance of oral microbiota alterations in PSCI. Overall, the present findings should be considered preliminary, exploratory and hypothesis-generating. Whether oral microbiota-based features can contribute to risk stratification or clinical decision-making remains unknown and requires validation in larger independent multicenter longitudinal cohorts.

## 5 Conclusion

Patients with post-stroke cognitive impairment exhibit oral microbiota dysbiosis, characterized by reduced richness and diversity together with altered community structure. Commensal taxa such as *Leptotrichia* and *Veillonella* are depleted, whereas Gram-negative lineages—including *Proteobacteria*, *Campylobacterota*, *Gammaproteobacteria*, and *Alloprevotella*—are enriched. These alterations may be associated with disruption of oral microbial homeostasis and may be relevant to cognitive impairment after stroke. In addition, several differentially abundant taxa showed exploratory associations with cognitive performance, and machine-learning models based on oral microbial features showed favorable internal discriminatory performance for distinguishing PSCI from PSNC within this dataset. However, these findings should be interpreted cautiously given the cross-sectional design, limited sample size, and lack of external validation. Overall, our results suggest that oral microbiota alterations are associated with PSCI and that selected oral microbial features may have exploratory internal discriminatory value within this dataset. These findings remain hypothesis-generating and require validation in larger multicenter longitudinal cohorts.

## Data Availability

The raw sequencing data generated in this study has been deposited in the NCBI Sequence Read Archive (SRA) under BioProject accession PRJNA1395059. The analysis code is publicly available at GitHub: https://github.com/Jianchao2022/R-code-in-PSCI.git.

## References

[ref1] Adil N. A. Omo-Erigbe C. Yadav H. Jain S. (2025). The oral-gut microbiome-brain axis in cognition. Microorganisms 13:814. doi: 10.3390/microorganisms13040814, 40284650 PMC12029813

[ref2] Allen G. C. Flores-Vergara M. A. Krasynanski S. Kumar S. Thompson W. F. (2006). A modified protocol for rapid DNA isolation from plant tissues using cetyltrimethylammonium bromide. Nat. Protoc. 1, 2320–2325. doi: 10.1038/nprot.2006.384, 17406474

[ref3] Arimatsu K. Yamada H. Miyazawa H. Minagawa T. Nakajima M. Ryder M. I. . (2014). Oral pathobiont induces systemic inflammation and metabolic changes associated with alteration of gut microbiota. Sci. Rep. 4:4828. doi: 10.1038/srep04828, 24797416 PMC4010932

[ref4] Bradshaw D. J. Marsh P. D. (1998). Analysis of pH-driven disruption of oral microbial communities *in vitro*. Caries Res. 32, 456–462. doi: 10.1159/000016487, 9745120

[ref5] Brown G. C. Heneka M. T. (2024). The endotoxin hypothesis of Alzheimer's disease. Mol. Neurodegener. 19:30. doi: 10.1186/s13024-024-00722-y, 38561809 PMC10983749

[ref6] Cho M. Y. Eom J. H. Choi E. M. Yang S. J. Lee D. Kim Y. Y. . (2025). Recent advances in therapeutic probiotics: insights from human trials. Clin. Microbiol. Rev. 38:e0024024. doi: 10.1128/cmr.00240-24, 40261032 PMC12160572

[ref7] Cho S. Jung Y. Oh H. S. Yum J. Song S. Jeong J. W. . (2025). Oral and gut dysbiosis in migraine: oral microbial signatures as biomarkers of migraine. Neurol Neuroimmunol. Neuroinflamm. 12:e200437. doi: 10.1212/nxi.0000000000200437, 40699951 PMC12221161

[ref8] Clasen F. Yildirim S. Arıkan M. Garcia-Guevara F. Hanoğlu L. Yılmaz N. H. . (2025). Microbiome signatures of virulence in the oral-gut-brain axis influence Parkinson's disease and cognitive decline pathophysiology. Gut Microbes 17:2506843. doi: 10.1080/19490976.2025.2506843, 40420833 PMC12118390

[ref9] Corrêa J. D. Faria G. A. Fernandes L. L. (2023). The oral microbiota and gestational diabetes mellitus. Front. Clin. Diabetes Healthc. 4:1120920. doi: 10.3389/fcdhc.2023.1120920, 36993820 PMC10012133

[ref10] Couturier M. R. Slechta E. S. Goulston C. Fisher M. A. Hanson K. E. (2012). Leptotrichia bacteremia in patients receiving high-dose chemotherapy. J. Clin. Microbiol. 50, 1228–1232. doi: 10.1128/jcm.05926-11, 22205794 PMC3318514

[ref11] Curtis M. A. Diaz P. I. Van Dyke T. E. (2020). The role of the microbiota in periodontal disease. Periodontol. 83, 14–25. doi: 10.1111/prd.12296, 32385883

[ref12] Curtis M. A. Zenobia C. Darveau R. P. (2011). The relationship of the oral microbiotia to periodontal health and disease. Cell Host Microbe 10, 302–306. doi: 10.1016/j.chom.2011.09.008, 22018230 PMC3216488

[ref13] Delwiche E. A. Pestka J. J. Tortorello M. L. (1985). The veillonellae: gram-negative cocci with a unique physiology. Ann. Rev. Microbiol. 39, 175–193. doi: 10.1146/annurev.mi.39.100185.001135, 3904599

[ref14] Deo P. N. Deshmukh R. (2019). Oral microbiome: unveiling the fundamentals. J. Oral Maxillofac. Pathol. 23, 122–128. doi: 10.4103/jomfp.JOMFP_304_18, 31110428 PMC6503789

[ref15] Douiri A. Rudd A. G. Wolfe C. D. (2013). Prevalence of poststroke cognitive impairment: South London stroke register 1995-2010. Stroke 44, 138–145. doi: 10.1161/strokeaha.112.670844, 23150656

[ref16] Edgar R. C. Haas B. J. Clemente J. C. Quince C. Knight R. (2011). UCHIME improves sensitivity and speed of chimera detection. Bioinformatics 27, 2194–2200. doi: 10.1093/bioinformatics/btr381, 21700674 PMC3150044

[ref17] Eribe E. R. Olsen I. (2008). *Leptotrichia* species in human infections. Anaerobe 14, 131–137. doi: 10.1016/j.anaerobe.2008.04.004, 18539056

[ref18] Eribe E. R. K. Olsen I. (2017). *Leptotrichia* species in human infections II. J. Oral Microbiol. 9:1368848. doi: 10.1080/20002297.2017.1368848, 29081911 PMC5646626

[ref19] Fu Y. Zhao J. Wang Y. (2025). LASSO regression and Boruta algorithm to explore the relationship between neutrophil percentage to albumin ratio and asthma: results from the NHANES 2001 to 2018. Clin. Exp. Med. 25:149. doi: 10.1007/s10238-025-01701-3, 40347409 PMC12065745

[ref20] Gao L. Xu T. Huang G. Jiang S. Gu Y. Chen F. (2018). Oral microbiomes: more and more importance in oral cavity and whole body. Protein Cell 9, 488–500. doi: 10.1007/s13238-018-0548-1, 29736705 PMC5960472

[ref21] Gaynor E. Rohde D. Large M. Mellon L. Hall P. Brewer L. . (2018). Cognitive impairment, vulnerability, and mortality post ischemic stroke: a five-year follow-up of the action on secondary prevention interventions and rehabilitation in stroke (ASPIRE-S) cohort. J. Stroke Cerebrovasc. Dis. 27, 2466–2473. doi: 10.1016/j.jstrokecerebrovasdis.2018.05.002, 29803601

[ref22] GBD 2021 Causes of Death Collaborators (2024). Global burden of 288 causes of death and life expectancy decomposition in 204 countries and territories and 811 subnational locations, 1990-2021: a systematic analysis for the global burden of disease study 2021. Lancet 403, 2100–2132. doi: 10.1016/s0140-6736(24)00367-2, 38582094 PMC11126520

[ref23] Giacomini J. J. Torres-Morales J. Dewhirst F. E. Borisy G. G. Mark Welch J. L. (2023). Site specialization of human oral *Veillonella* species. Microbiol. Spectrum 11:e0404222. doi: 10.1128/spectrum.04042-22, 36695592 PMC9927086

[ref24] Haas B. J. Gevers D. Earl A. M. Feldgarden M. Ward D. V. Giannoukos G. . (2011). Chimeric 16S rRNA sequence formation and detection in sanger and 454-pyrosequenced PCR amplicons. Genome Res. 21, 494–504. doi: 10.1101/gr.112730.110, 21212162 PMC3044863

[ref25] Han Y. W. Wang X. (2013). Mobile microbiome: oral bacteria in extra-oral infections and inflammation. J. Dent. Res. 92, 485–491. doi: 10.1177/0022034513487559, 23625375 PMC3654760

[ref26] Hu J. Szymczak S. (2023). A review on longitudinal data analysis with random forest. Brief. Bioinform. 24:bbad002. doi: 10.1093/bib/bbad002, 36653905 PMC10025446

[ref27] Huang Y. Y. Chen S. D. Leng X. Y. Kuo K. Wang Z.-T. Cui M. . (2022). Post-stroke cognitive impairment: epidemiology, risk factors, and management. J. Alzheimer's Dis 86, 983–999. doi: 10.3233/jad-215644, 35147548

[ref28] Jørgensen S. F. Trøseid M. Kummen M. Anmarkrud J. A. Michelsen A. E. Osnes L. T. . (2016). Altered gut microbiota profile in common variable immunodeficiency associates with levels of lipopolysaccharide and markers of systemic immune activation. Mucosal Immunol. 9, 1455–1465. doi: 10.1038/mi.2016.18, 26982597

[ref29] Katoh K. Misawa K. Kuma K. Miyata T. (2002). MAFFT: a novel method for rapid multiple sequence alignment based on fast Fourier transform. Nucleic Acids Res. 30, 3059–3066. doi: 10.1093/nar/gkf436, 12136088 PMC135756

[ref30] Khleborodova A. Gamboa-Tuz S. D. Ramos M. Segata N. Waldron L. Oh S. (2024). Lefser: implementation of metagenomic biomarker discovery tool, LEfSe, in R. Bioinformatics 40:btae707. doi: 10.1093/bioinformatics/btae707, 39585730 PMC11665633

[ref31] Langfeldt D. Neulinger S. C. Stiesch M. Stumpp N. Bang C. Schmitz R. A. . (2014). Health- and disease-associated species clusters in complex natural biofilms determine the innate immune response in oral epithelial cells during biofilm maturation. FEMS Microbiol. Lett. 360, 137–143. doi: 10.1111/1574-6968.12596, 25212593

[ref32] LaValley M. P. (2008). Logistic regression. Circulation 117, 2395–2399. doi: 10.1161/circulationaha.106.682658, 18458181

[ref33] Lecca D. Jung Y. J. Scerba M. T. Hwang I. Kim Y. K. Kim S. . (2022). Role of chronic neuroinflammation in neuroplasticity and cognitive function: a hypothesis. Alzheimers Dement. 18, 2327–2340. doi: 10.1002/alz.12610, 35234334 PMC9437140

[ref34] Leonov G. Salikhova D. Starodubova A. Vasilyev A. Makhnach O. Fatkhudinov T. . (2024). Oral microbiome Dysbiosis as a risk factor for stroke: a comprehensive review. Microorganisms 12:1732. doi: 10.3390/microorganisms12081732, 39203574 PMC11357103

[ref35] Li D. Ren T. Li H. Huang M. Chen J. He Q. . (2024). Oral microbiota and *Porphyromonas gingivalis* Kgp genotypes altered in Parkinson's disease with mild cognitive impairment. Mol. Neurobiol. 61, 8631–8639. doi: 10.1007/s12035-024-04119-2, 38536604

[ref36] Liang J. Ren Y. Zheng Y. Lin X. Song W. Zhu J. . (2025). Functional outcome prediction of acute ischemic stroke based on the Oral and gut microbiota. Mol. Neurobiol. 62, 5413–5431. doi: 10.1007/s12035-024-04618-2, 39546118 PMC11953115

[ref37] Listgarten M. A. (1994). The structure of dental plaque. Periodontol. 5, 52–65. doi: 10.1111/j.1600-0757.1994.tb00018.x, 9673162

[ref38] Litvak Y. Byndloss M. X. Tsolis R. M. Bäumler A. J. (2017). Dysbiotic Proteobacteria expansion: a microbial signature of epithelial dysfunction. Curr. Opin. Microbiol. 39, 1–6. doi: 10.1016/j.mib.2017.07.003, 28783509

[ref39] Liu X. X. Jiao B. Liao X. X. Guo L. N. Yuan Z. H. Wang X. . (2019). Analysis of salivary microbiome in patients with Alzheimer's disease. J. Alzheimer's Dis 72, 633–640. doi: 10.3233/jad-190587, 31594229

[ref40] Lo J. W. Crawford J. D. Desmond D. W. Godefroy O. Jokinen H. Mahinrad S. . (2019). Profile of and risk factors for poststroke cognitive impairment in diverse ethnoregional groups. Neurology 93, e2257–e2271. doi: 10.1212/wnl.0000000000008612, 31712368 PMC6937495

[ref41] Ma X. Kim J. K. Shin Y. J. Park H. S. Lee D. Y. Yim S. V. . (2024). Lipopolysaccharide-producing Veillonella infantium and *Escherichia fergusonii* cause vagus nerve-mediated cognitive impairment in mice. Brain Behav. Immun. 118, 136–148. doi: 10.1016/j.bbi.2024.02.031, 38428648

[ref42] Ma B. Meng F. Yan G. Yan H. Chai B. Song F. (2020). Diagnostic classification of cancers using extreme gradient boosting algorithm and multi-omics data. Comput. Biol. Med. 121:103761. doi: 10.1016/j.compbiomed.2020.103761, 32339094

[ref43] Magoč T. Salzberg S. L. (2011). FLASH: fast length adjustment of short reads to improve genome assemblies. Bioinformatics 27, 2957–2963. doi: 10.1093/bioinformatics/btr507, 21903629 PMC3198573

[ref44] Mahon S. Parmar P. Barker-Collo S. Krishnamurthi R. Jones K. Theadom A. . (2017). Determinants, prevalence, and trajectory of long-term post-stroke cognitive impairment: results from a 4-year follow-up of the ARCOS-IV study. Neuroepidemiology 49, 129–134. doi: 10.1159/000484606, 29145207

[ref45] Mehrpour O. Hoyte C. Nakhaee S. Megarbane B. Goss F. (2023). Using a decision tree algorithm to distinguish between repeated supra-therapeutic and acute acetaminophen exposures. BMC Med. Inform. Decis. Mak. 23:102. doi: 10.1186/s12911-023-02188-2, 37264381 PMC10233916

[ref46] Mijajlović M. D. Pavlović A. Brainin M. Heiss W. D. Quinn T. J. Ihle-Hansen H. B. . (2017). Post-stroke dementia - a comprehensive review. BMC Med. 15:11. doi: 10.1186/s12916-017-0779-7, 28095900 PMC5241961

[ref47] Mohammad S. Thiemermann C. (2020). Role of metabolic endotoxemia in systemic inflammation and potential interventions. Front. Immunol. 11:594150. doi: 10.3389/fimmu.2020.594150, 33505393 PMC7829348

[ref48] Narengaowa Kong W. Lan F. Awan U. F. Qing H. Ni J. (2021). The oral-gut-brain AXIS: the influence of microbes in Alzheimer's disease. Front. Cell. Neurosci. 15:633735. doi: 10.3389/fncel.2021.633735 33935651 PMC8079629

[ref49] Nasreddine Z. S. Phillips N. A. Bédirian V. Charbonneau S. Whitehead V. Collin I. . (2005). The Montreal cognitive assessment, MoCA: a brief screening tool for mild cognitive impairment. J. Am. Geriatr. Soc. 53, 695–699. doi: 10.1111/j.1532-5415.2005.53221.x, 15817019

[ref50] Ng S. K. Hamilton I. R. (1971). Lactate metabolism by *Veillonella parvula*. J. Bacteriol. 105, 999–1005. doi: 10.1128/jb.105.3.999-1005.1971, 4323300 PMC248529

[ref51] Nonaka S. Nakanishi H. (2020). Secreted gingipains from *Porphyromonas gingivalis* induce microglia migration through endosomal signaling by protease-activated receptor 2. Neurochem. Int. 140:104840. doi: 10.1016/j.neuint.2020.104840, 32858090

[ref52] Qu Y. Zhuo L. Li N. Hu Y. Chen W. Zhou Y. . (2015). Prevalence of post-stroke cognitive impairment in China: a community-based, cross-sectional study. PLoS One 10:e0122864. doi: 10.1371/journal.pone.0122864, 25874998 PMC4395303

[ref53] Quast C. Pruesse E. Yilmaz P. Gerken J. Schweer T. Yarza P. . (2013). The SILVA ribosomal RNA gene database project: improved data processing and web-based tools. Nucleic Acids Res. 41, D590–D596. doi: 10.1093/nar/gks1219 23193283 PMC3531112

[ref54] Raetz C. R. Whitfield C. (2002). Lipopolysaccharide endotoxins. Annu. Rev. Biochem. 71, 635–700. doi: 10.1146/annurev.biochem.71.110601.135414, 12045108 PMC2569852

[ref55] Ren Z. Cui G. Lu H. Chen X. Jiang J. Liu H. . (2013). Liver ischemic preconditioning (IPC) improves intestinal microbiota following liver transplantation in rats through 16s rDNA-based analysis of microbial structure shift. PLoS One 8:e75950. doi: 10.1371/journal.pone.0075950, 24098410 PMC3788797

[ref56] Ren Y. Liang J. Li X. Deng Y. Cheng S. Wu Q. . (2023). Association between oral microbial dysbiosis and poor functional outcomes in stroke-associated pneumonia patients. BMC Microbiol. 23:305. doi: 10.1186/s12866-023-03057-8, 37875813 PMC10594709

[ref57] Reyes L. (2021). Porphyromonas gingivalis. Trends Microbiol. 29, 376–377. doi: 10.1016/j.tim.2021.01.010, 33546976

[ref58] Rizzatti G. Lopetuso L. R. Gibiino G. Binda C. Gasbarrini A. (2017). Proteobacteria: a common factor in human diseases. Biomed. Res. Int. 2017, 1–7. doi: 10.1155/2017/9351507, 29230419 PMC5688358

[ref59] Rozas N. S. Tribble G. D. Jeter C. B. (2021). Oral factors that impact the Oral microbiota in Parkinson's disease. Microorganisms 9:1616. doi: 10.3390/microorganisms9081616, 34442695 PMC8402101

[ref60] Sandiego C. M. Gallezot J. D. Pittman B. Nabulsi N. Lim K. Lin S. F. . (2015). Imaging robust microglial activation after lipopolysaccharide administration in humans with PET. Proc. Natl. Acad. Sci. USA 112, 12468–12473. doi: 10.1073/pnas.1511003112, 26385967 PMC4603509

[ref61] Sansores-España L. D. Melgar-Rodríguez S. Olivares-Sagredo K. Cafferata E. A. Martínez-Aguilar V. M. Vernal R. . (2021). Oral-gut-brain Axis in experimental models of periodontitis: associating gut Dysbiosis with neurodegenerative diseases. Front. Aging 2:781582. doi: 10.3389/fragi.2021.781582, 35822001 PMC9261337

[ref62] Segata N. Haake S. K. Mannon P. Lemon K. P. Waldron L. Gevers D. . (2012). Composition of the adult digestive tract bacterial microbiome based on seven mouth surfaces, tonsils, throat and stool samples. Genome Biol. 13:R42. doi: 10.1186/gb-2012-13-6-r42, 22698087 PMC3446314

[ref63] Sender R. Fuchs S. Milo R. (2016a). Are we really vastly outnumbered? Revisiting the ratio of bacterial to host cells in humans. Cell 164, 337–340. doi: 10.1016/j.cell.2016.01.013, 26824647

[ref64] Sender R. Fuchs S. Milo R. (2016b). Revised estimates for the number of human and Bacteria cells in the body. PLoS Biol. 14:e1002533. doi: 10.1371/journal.pbio.1002533, 27541692 PMC4991899

[ref65] Sexton E. McLoughlin A. Williams D. J. Merriman N. A. Donnelly N. Rohde D. . (2019). Systematic review and meta-analysis of the prevalence of cognitive impairment no dementia in the first year post-stroke. Eur. Stroke J. 4, 160–171. doi: 10.1177/2396987318825484, 31259264 PMC6591758

[ref66] Skrzypczak-Wiercioch A. Sałat K. (2022). Lipopolysaccharide-induced model of Neuroinflammation: mechanisms of action, research application and future directions for its use. Molecules 27:5481. doi: 10.3390/molecules27175481, 36080253 PMC9457753

[ref67] Sureda A. Daglia M. Argüelles Castilla S. Sanadgol N. Fazel Nabavi S. Khan H. . (2020). Oral microbiota and Alzheimer's disease: do all roads lead to Rome? Pharmacol. Res. 151:104582. doi: 10.1016/j.phrs.2019.104582, 31794871

[ref68] Tett A. Pasolli E. Masetti G. Ercolini D. Segata N. (2021). Prevotella diversity, niches and interactions with the human host. Nat. Rev. Microbiol. 19, 585–599. doi: 10.1038/s41579-021-00559-y, 34050328 PMC11290707

[ref69] Valkenborg D. Rousseau A. J. Geubbelmans M. Burzykowski T. (2023). Support vector machines. Am. J. Orthod. Dentofacial Orthop. 164, 754–757. doi: 10.1016/j.ajodo.2023.08.003, 37914440

[ref70] Washio J. Shimada Y. Yamada M. Sakamaki R. Takahashi N. (2014). Effects of pH and lactate on hydrogen sulfide production by oral Veillonella spp. Appl. Environ. Microbiol. 80, 4184–4188. doi: 10.1128/aem.00606-14, 24795374 PMC4068660

[ref71] Wu Y. F. Lee W. F. Salamanca E. Yao W. L. Su J. N. Wang S. Y. . (2021). Oral microbiota changes in elderly patients, an Indicator of Alzheimer's disease. Int. J. Environ. Res. Public Health 18:4211. doi: 10.3390/ijerph18084211, 33921182 PMC8071516

[ref72] Zangeneh Z. Abdi-Ali A. Khamooshian K. Alvandi A. Abiri R. (2021). Bacterial variation in the oral microbiota in multiple sclerosis patients. PLoS One 16:e0260384. doi: 10.1371/journal.pone.0260384, 34847159 PMC8631616

[ref73] Zhang Z. (2016). Introduction to machine learning: k-nearest neighbors. Ann. Transl. Med. 4:218. doi: 10.21037/atm.2016.03.37, 27386492 PMC4916348

[ref74] Zhang J. Mucs D. Norinder U. Svensson F. (2019). LightGBM: an effective and scalable algorithm for prediction of chemical toxicity-application to the Tox21 and mutagenicity data sets. J. Chem. Inf. Model. 59, 4150–4158. doi: 10.1021/acs.jcim.9b00633, 31560206

[ref75] Zhang Z. Wang J. Han W. Zhao L. (2023). Using machine learning methods to predict 28-day mortality in patients with hepatic encephalopathy. BMC Gastroenterol. 23:111. doi: 10.1186/s12876-023-02753-z, 37024814 PMC10077693

[ref76] Zhong Y. Kang X. Bai X. Pu B. Smerin D. Zhao L. . (2024). The Oral-gut-brain Axis: the influence of microbes as a link of periodontitis with ischemic stroke. CNS Neurosci. Ther. 30:e70152. doi: 10.1111/cns.70152, 39675010 PMC11646473

