# Supplementary material for: Oral microbiome signatures of post-stroke cognitive impairment
Source: Front Microbiol. 2026 Jun 5;17:1722999. doi: 10.3389/fmicb.2026.1722999 (PMC13278991; doi:10.3389/fmicb.2026.1722999)
Supplement: Supplementary file 1 [file Table_1.DOCX]

Supplementary Material

# Supplementary Tables

***Supplementary Table S1. Multivariable-adjusted associations between PSCI and alpha diversity indices***

| Model | β | 95% CI | *P* value | VIF |
| --- | --- | --- | --- | --- |
| Shannon ~ Group + NIHSS + BBS + BI | -0.666 | -1.198 – -0.134 | **0.0148** | 1.66 |
| Simpson ~ Group + NIHSS + BBS + BI | -0.097 | -0.194 – -0.002 | **0.0457** | 1.66 |
| Chao1 ~ Group + NIHSS + BBS + BI | -69.756 | -109.149 – -30.365 | **<0.001** | 1.66 |
| ACE ~ Group + NIHSS + BBS + BI | -69.855 | -109.283 – -30.427 | **<0.001** | 1.66 |

Linear regression models were adjusted for NIHSS score, Berg Balance Scale (BBS), and Activities of Daily Living (BI). β represents regression coefficients for each predictor. VIF indicates variance inflation factors used to assess multicollinearity.

***Supplementary Table S2. Covariate-adjusted PERMANOVA results***

| Distance | Variable | R² | F | *P* value |
| --- | --- | --- | --- | --- |
| Bray–Curtis | Group | 0.0199 | 1.632 | **0.015** |
|  | NIHSS | 0.0118 | 0.962 | 0.512 |
|  | BBS | 0.0141 | 1.151 | 0.302 |
|  | ADL | 0.0109 | 0.893 | 0.601 |

Covariate-adjusted beta diversity differences were evaluated by PERMANOVA (adonis2, vegan) on Bray–Curtis distances with NIHSS, BBS, and ADL as covariates (999 permutations).

***Supplementary Table S3. PERMDISP (betadisper) results based on Bray–Curtis distances***

| Distance | Term | Df | F | Permutations | *P* value |
| --- | --- | --- | --- | --- | --- |
| Bray–Curtis | Groups | 1 | 1.893 | 999 | 0.167 |

The homogeneity of multivariate dispersion among groups was assessed using PERMDISP implemented in the **betadisper** function (vegan package) with 999 permutations. No significant difference in within-group dispersion was observed (*P* = 0.167), indicating that the significant PERMANOVA results were unlikely to be driven by differences in group dispersion.

***Supplementary Table S4. Performance of eight machine learning models in the test set***

| **Model** | **AUC (95% CI)** | **AP (95% CI)** | **Sensitivity** | **Specificity** | **Accuracy** | **Balanced accuracy** | **PPV** | **NPV** | **F1** | **Brier score** | **LogLoss** |
| --- | --- | --- | --- | --- | --- | --- | --- | --- | --- | --- | --- |
| **Random Forest** | 0.979 (0.917-1.000) | 0.983 (0.931-1.000) | 0.917 | 1.000 | 0.958 | 0.958 | 1.000 | 0.923 | 0.957 | 0.076 | 0.256 |
| **Neural Network** | 0.979 (0.917-1.000) | 0.978 (0.920-1.000) | 0.667 | 1.000 | 0.833 | 0.833 | 1.000 | 0.750 | 0.800 | 0.127 | 0.358 |
| **XGBoost** | 0.972 (0.903-1.000) | 0.976 (0.915-1.000) | 0.750 | 1.000 | 0.875 | 0.875 | 1.000 | 0.800 | 0.857 | 0.089 | 0.317 |
| **LightGBM** | 0.958 (0.861-1.000) | 0.965 (0.893-1.000) | 0.917 | 0.750 | 0.833 | 0.833 | 0.786 | 0.900 | 0.846 | 0.099 | 0.359 |
| **SVM** | 0.889 (0.736-0.986) | 0.909 (0.787-0.988) | 0.500 | 1.000 | 0.750 | 0.750 | 1.000 | 0.667 | 0.667 | 0.150 | 0.465 |
| **Decision Tree** | 0.875 (0.750-1.000) | 0.918 (0.821-1.000) | 0.750 | 1.000 | 0.875 | 0.875 | 1.000 | 0.800 | 0.857 | 0.103 | 0.347 |
| **Logistic Regression** | 0.812 (0.604-1.000) | 0.887 (0.745-1.000) | 0.667 | 1.000 | 0.833 | 0.833 | 1.000 | 0.750 | 0.800 | 0.158 | 0.738 |
| **KNN** | 0.792 (0.625-0.958) | 0.804 (0.612-0.946) | 0.667 | 0.917 | 0.792 | 0.792 | 0.889 | 0.733 | 0.762 | 0.208 | 2.878 |

The performance of eight machine learning models was evaluated in the test set. AUC indicates the area under the receiver operating characteristic curve; AP, average precision, corresponding to the area under the precision–recall curve; CI, confidence interval; PPV, positive predictive value; NPV, negative predictive value; F1, F1 score; LogLoss, logarithmic loss; KNN, k-nearest neighbors; SVM, support vector machine. The 95% confidence intervals for AUC and AP were estimated using 2000 bootstrap resampling. Sensitivity, specificity, accuracy, balanced accuracy, PPV, NPV, and F1 were calculated based on the classification results in the test set. Brier score and LogLoss were used to assess the quality of probabilistic predictions, with lower values indicating better calibration and probability prediction performance.

***Supplementary Table S5. Repeated cross-validation stability of the random forest model in the training set***

| **Metric** | **Mean ± SD** | **Median** | **Empirical 95% interval** |
| --- | --- | --- | --- |
| **AUC** | 0.960 ± 0.052 | 0.986 | 0.840-1.000 |
| **AP** | 0.969 ± 0.038 | 0.987 | 0.875-1.000 |
| **Sensitivity** | 0.903 ± 0.114 | 1.000 | 0.667-1.000 |
| **Specificity** | 0.892 ± 0.131 | 1.000 | 0.600-1.000 |
| **Accuracy** | 0.897 ± 0.085 | 0.909 | 0.682-1.000 |
| **Balanced accuracy** | 0.898 ± 0.085 | 0.917 | 0.682-1.000 |
| **PPV** | 0.905 ± 0.111 | 1.000 | 0.667-1.000 |
| **NPV** | 0.912 ± 0.103 | 1.000 | 0.689-1.000 |
| **F1** | 0.897 ± 0.085 | 0.909 | 0.667-1.000 |
| **Brier score** | 0.080 ± 0.047 | 0.073 | 0.016-0.189 |
| **LogLoss** | 0.274 ± 0.127 | 0.249 | 0.097-0.552 |

The internal stability of the random forest model was assessed using repeated cross-validation within the training set. The values represent the distribution of model performance across repeated resampling procedures. AUC indicates the area under the receiver operating characteristic curve; AP, average precision; PPV, positive predictive value; NPV, negative predictive value; F1, F1 score; LogLoss, logarithmic loss. The empirical 95% interval was defined as the 2.5th to 97.5th percentiles of the metric distribution across repeated cross-validation runs. Higher values of AUC, AP, sensitivity, specificity, accuracy, balanced accuracy, PPV, NPV, and F1 indicate better model performance, whereas lower Brier score and LogLoss indicate better probabilistic prediction performance.

***Supplementary Table S6. Stability of selected microbial taxa across bootstrap resampling***

| **Taxon** | **Original LASSO** | **Original RF rank** | **Original RF MGI** | **LASSO frequency, %** | **RF frequency, %** | **Intersection frequency, %** | **RF Top1, %** | **RF Top2, %** | **RF Top3, %** | **RF MGI, mean ± SD** | **RF rank, median (IQR)** |
| --- | --- | --- | --- | --- | --- | --- | --- | --- | --- | --- | --- |
| **g__Leptotrichia** | Yes | 1 | 11.553 | 99.9 | 100.0 | 99.9 | 74.2 | 99.4 | 100.0 | 11.186 ± 1.187 | 1 (1) |
| **g__Veillonella** | Yes | 2 | 9.753 | 99.5 | 100.0 | 99.5 | 25.1 | 91.9 | 100.0 | 9.670 ± 1.380 | 2 (1) |
| **g__Campylobacter** | Yes | 3 | 6.200 | 96.5 | 100.0 | 96.5 | 0.7 | 8.7 | 100.0 | 6.643 ± 1.159 | 3 (0) |

The stability of selected microbial taxa was assessed using bootstrap resampling within the training set. “Original LASSO” indicates whether the taxon was selected by LASSO in the original training-set analysis. “Original RF rank” and “Original RF MGI” indicate the rank and mean decrease Gini importance of each taxon in the original random forest model. “LASSO frequency” represents the proportion of bootstrap resamples in which the taxon was selected by LASSO. “RF frequency” represents the proportion of bootstrap resamples in which the taxon was retained by the random forest model. “Intersection frequency” represents the proportion of bootstrap resamples in which the taxon was simultaneously selected by both LASSO and random forest. “RF Top1,” “RF Top2,” and “RF Top3” indicate the proportion of bootstrap resamples in which the taxon ranked within the top 1, top 2, or top 3 features according to random forest importance. RF MGI indicates random forest means decrease Gini importance; IQR indicates interquartile range.
